# Dietary Alleviation of Maternal Obesity and Diabetes: Increased Resistance to Diet-Induced Obesity Transcriptional and Epigenetic Signatures

**DOI:** 10.1371/journal.pone.0066816

**Published:** 2013-06-24

**Authors:** Linda Attig, Alexandre Vigé, Anne Gabory, Moshen Karimi, Aurore Beauger, Marie-Sylvie Gross, Anne Athias, Catherine Gallou-Kabani, Philippe Gambert, Tomas J. Ekstrom, Jean-Philippe Jais, Claudine Junien

**Affiliations:** 1 INRA, UMR1198 Biologie du Développement et Reproduction, Jouy-en-Josas, France; 2 INSERM U781 AP-HP; Université Paris-Descartes, Faculté de Médecine, Hôpital Necker-Enfants, Paris, France; 3 Laboratory for Medical Epigenetics, Center for Molecular Medicine, Department of Clinical Neuroscience, Karolinska Institutet, Stockholm, Sweden; 4 IFR100 Santé-STIC, Plateau Technique Lipidomique, CHU Bocage Bat B2, Dijon, France; 5 IFR100 Santé-STIC, Laboratoire de Biochimie Médicale, Plateau Technique de Biologie, Dijon, France; 6 Service de Biostatistique et Informatique Médicale, Université Paris Descartes, Hôpital Necker-Enfants Malades, Paris, France; Blaise Pascal University, France

## Abstract

According to the developmental origins of health and diseases (DOHaD), and in line with the findings of many studies, obesity during pregnancy is clearly a threat to the health and well-being of the offspring, later in adulthood. We previously showed that 20% of male and female inbred mice can cope with the obesogenic effects of a high-fat diet (HFD) for 20 weeks after weaning, remaining lean. However the feeding of a control diet (CD) to DIO mice during the periconceptional/gestation/lactation period led to a pronounced sex-specific shift (17% to 43%) from susceptibility to resistance to HFD, in the female offspring only. Our aim in this study was to determine how, in the context of maternal obesity and T2D, a CD could increase resistance on female fetuses. Transcriptional analyses were carried out with a custom-built mouse liver microarray and by quantitative RT-PCR for muscle and adipose tissue. Both global DNA methylation and levels of pertinent histone marks were assessed by LUMA and western blotting, and the expression of 15 relevant genes encoding chromatin-modifying enzymes was analyzed in tissues presenting global epigenetic changes. Resistance was associated with an enhancement of hepatic pathways protecting against steatosis, the unexpected upregulation of neurotransmission-related genes and the modulation of a vast imprinted gene network. Adipose tissue displayed a pronounced dysregulation of gene expression, with an upregulation of genes involved in lipid storage and adipocyte hypertrophy or hyperplasia in obese mice born to lean and obese mothers, respectively. Global DNA methylation, several histone marks and key epigenetic regulators were also altered. Whether they were themselves lean (resistant) or obese (sensitive), the offspring of lean and obese mice clearly differed in terms of several metabolic features and epigenetic marks suggesting that the effects of a HFD depend on the leanness or obesity of the mother.

## Introduction

The incidence of non-communicable diseases (NCDs), which already account for 60% of deaths worldwide, is expected to increase by 17% in the next decade (WHO). However the fundamental misconceptions associated with the current focus of action on obesity and NCDs call for a paradigm shift, incorporating a new dimension: the developmental origins of health and diseases (DOHaD). Indeed, early nutritional events may influence health in later life, mostly through epigenetic processes. In genetically identical mice, some individuals are resistant to diet-induced obesity, whereas most display variable degrees of diet-induced obesity (DIO) and/or type 2 diabetes (T2D), with different patterns of metabolic adaptation, even if maintained in seemingly identical environmental conditions. The reasons for this remain unclear. Likewise, despite the worldwide increase in obesity and related diseases, most individuals are neither overweight nor obese, and are obviously able to maintain a balance between dietary intake and energy expenditure, leaving them better “armed” than others to deal with the plethora of food on offer. According to the DOHaD concept, environmental conditions during specific windows of mammalian development can have lasting effects on cell fate, organogenesis, metabolic pathways and physiology, thereby influencing life-long physical health and the susceptibility to lifestyle-induced diseases in adulthood [1], [2]. There is evidence to suggest that maternal overnutrition, gestational diabetes and obesity are deleterious to the health of offspring, inducing the same range of defects as maternal mal- or undernutrition and leading to the development of metabolic syndrome [3], [4], [5], [6], [7] in the offspring, with a striking sex-specificity [8], [9], [10]. The number of overweight or obese women of child-bearing age is growing, and has reached 25% in Europe and 50% in the US (WHO). This could trigger a vicious cycle, with transmission to subsequent generations and an increasing prevalence of these lifestyle-induced disorders. Interestingly, the adverse metabolic consequences of dietary manipulations can be improved or prevented by applying mild food restriction or a normal control diet to the mother [11], [12], by reducing maternal obesity by bariatric surgery [13], [14] or by the addition to the maternal diet of specific nutrients involved in various levels of carbon metabolism essential for DNA methylation [15], [16], [17], [18], [19], [20].

We previously showed that 83% of F1 females (F1LM), born to F0 CD-fed lean mothers develop hyperphagia, obesity and T2D in response to a post-weaning high-fat diet (HFD) for 20 weeks, but with 17% remaining lean, with normal insulin sensitivity, despite the HFD. When F1 females with diet-induced obesity (DIO) and T2D (F2OM) were fed a CD with an appropriate dietary fatty-acid profile during the periconceptional/gestation/lactation period, we observed a strong shift toward resistance (increasing from 17% to 43%) in their offspring fed an obesogenic diet after weaning. However, this shift in sensitivity was restricted to females [21]. This sex-specificity is frequently found in studies of developmental programming [8], [9], [10], [22]. Thus, a CD can alleviate the malprogramming effects of maternal obesity and type 2 diabetes (T2D) in mice, in a sex-specific manner, probably through adaptation to adverse intrauterine conditions.

In this study, focusing on the female offspring born to either lean mothers (F1LM or F1) or to obese mothers (F2OM or F2) fed a CD during the periconceptional/gestation/lactation period, we investigated phenotypic and metabolic features, the transcriptional and epigenetic mechanisms underlying the metabolic response and adaptation to HFD, and the trait of resistance/susceptibility to the obesogenic effects of a HFD. We used a candidate gene approach to study categories of genes likely to be affected by lipid overload and to contribute to the hepatic response to HFD and long-term adaptation. A custom-built microarray [23] was used for the transcriptional analysis of: 1) classical candidate genes controlling metabolism, 2) genes regulated by genomic imprinting potentially susceptible to nutritional disturbances [24], [25], [26], and, 3) genes involved in neurotransmission of potential importance for the control of hepatic function and energy status in the liver [27], [28], [29]. Quantitative RT-PCR (RT-qPCR) experiments were also carried out in the adipose tissue, which plays a key role in lipid storage. Global epigenetic modifications were assessed, by LUMA for DNA methylation and western blotting for pertinent histone marks, to identify changes in response to diet as a function of resistance status. We then investigated the relationship between these modifications and potential changes in enzymatic activities, by analyzing the expression of 15 genes encoding chromatin-modifying enzymes in tissues presenting global epigenetic changes, by RT-qPCR.

## Results and Discussion

### Phenotypic and Metabolic Data

**Figure 1:**
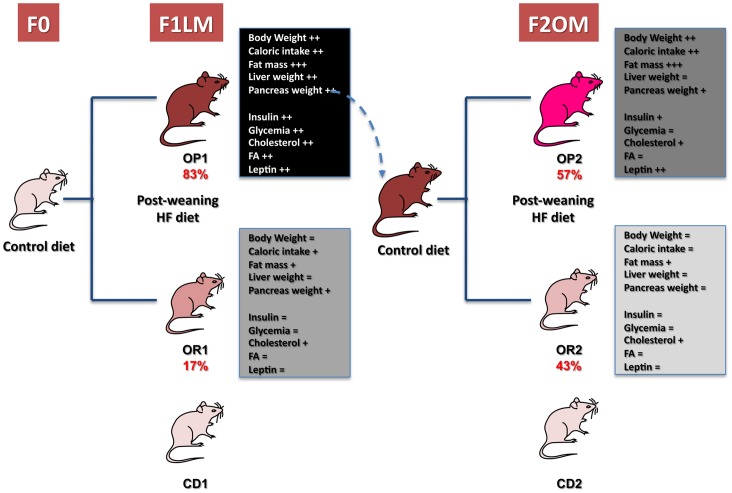
Schematic diagram summarizing the step-by-step evolution of the metabolic phenotype of OR and OP mice in the F1LM and F2OM offspring.

As previously described, the offspring of lean control or obese, diabetic female mice fed a CD during the periconceptional/gestation/lactation period were fed a HFD for five months after weaning [21]. Figure 1 summarizes all the metabolic data. Mice with body weights more than two standard deviations above the mean weight of the controls were classified as obesity-prone (OP), whereas mice with body weights below this threshold were classified as obesity-resistant (OR). This highlights the step-by-step development of the metabolic phenotype, from mice prone to obesity (full phenotype) when challenged with an obesogenic diet after weaning (OP1), to obesity-resistant mice (OR2 mice) with an almost normal phenotype despite HFD-associated mild dyslipidemia. OP mice accounted for 83% (OP1) of the F1 daughters of the lean F0 mothers (F1LM) and for 57% (OP2) of the F2 daughters of the F1 obese and diabetic mothers (F2OM) and weighed, on average, 75% and 62% more than their respective controls (CD1, CD2) fed the control diet (CD) (Table 1). Obesity was generally associated with hyperphagia and high caloric intake in OP mice. However, 17% of the F1LM female offspring and 43% of the F2OM female offspring remained lean, with body weights similar to those of CD1 and CD2 mice (Figure 1), despite being fed the HFD. These mice were classified as obesity-resistant (OR1 and OR2 for the F1LM and F2OM, respectively; Figure 1). OR1 mice had a caloric intake slightly higher than that of CD1, but much lower than that of OP1 and OP2 mice. OR2 mice had a caloric intake similar to that for CD2 mice. Piximus technology and the dissection of adipose tissue from various locations showed that body fat content was much higher in OP1 and OP2 obese mice, reaching about 40% of body weight. OR1 and OR2 mice also had a slightly higher fat content than their respective controls, but this difference was much smaller than that for OP1 and OP2 mice (Table 1). OP1 mice had heavier livers than control mice, whereas OP2, OR1 and OR2 mice had liver weights similar to that in control mice (Figure 1). Severe pancreatic hypertrophy (tripling of organ weight) was also observed in OP1, OP2 and OR1 mice, but not in OR2 mice (Figure 1).

**Table 1:** Phenotypic and metabolic characteristics of the mice after 24 weeks on the CD and HFD, for the F1LM (CD1, OP1 and OR1) and F2OM (CD2, OP2 and OR2) mice.

Parameters	CD1	OP1	OR1	CD2	OP2	OR2
**Body composition and food intake**						
Weight (g)	21.35±0.12	37.28a,b±0.55	22.33±0.33	21.14±0.14	34.37a,b±1.09	21.5±0.28
Caloric intake (kcal/5mo)	1300±8	1856a,b±83	1451^a^±30	1283±19	1848a,b±58	1344±30
Fat mass (%)	18.42±1.12	42.34a,b±2.07	29.00^a^±1.32	15.61±0.63	40.46a,b±3.96	27.97^a^±2.2
**Organ weights**						
Heart (g)	0.123±0.013	0.142±0.009	0.142±0.019	0.155±0.016	0.142±0.014	0.109±0.009
Liver (g)	0.826±0.110	1.165a,b±0.068	0.902±0.063	0.785±0.120	0.902^c^±0.050	0.723±0.090
Spleen (g)	0.100±0.015	0.107±0.010	0.112±0.018	0.120±0.020	0.112±0.019	0.106±0.035
Pancreas (g)	0.343±0.023	1.195^a^±0.145	1.172^a^±0.164	0.387±0.011	1.172a,b±0.164	0.53±0.122
Epididymal AT (g)	0.176±0.014	1.851a,b±0.260	2.080^a^±0.588	0.256±0.045	2.552a,b±0.453	0.686^a^±0.301
Retroperitoneal AT (g)	0.123±0.016	1.602a,b±0.554	1.294^a^±0.397	0.112±0.043	1.583a,b±0.356	0.436^a^±0.173
Subcutaneous AT (g)	0.73±0.111	5.748a,b±1.103	3.968^a^±0.990	0.663±0.069	4.808a,b±0.679	1.223^a^±0.789
**Plasma hormones and metabolites**						
Insulin (ng/ml)	0.89±0.08	2.38a,b±0.31	0.77±0.10	0.99±0.08	1.48a,b,c±0.38	0.96±0.12
Leptin (ng/ml)	4.6±0.7	40.6a,b±4.1	8.2±2.4	–	34.0^b^±5.0	9.5±3.4
Glucose (mmol/l)	6.89±0.19	8.6a,b±0.26	7.1±0.3	6.54±0.21	8.1a,b,c±0.2	7.2±0.4
Cholesterol (mmol/l)	1.59±0.07	2.58a,b±0.15	2.17^a^±0.14	1.36±0.12	2.37a,b±0.26	2.00^a^±0.14
HDL (mmol/l)	1.03±0.03	1.61±0.08	1.53±0.09	0.89±0.09	1.28±0.16	1.20±0.06
**Circulating fatty acids**						
Total FA (µg/ml)	186.4±6.6	334.0^a^±22.1	256.7^a^±30.0	198.4±6.0	202.2^c^±14.8	174.0^c^±16.9
C16∶0 (µg/ml)	45.2±3.4	36.8±3.0	39.9±7.3	34.5±4.8	26.1±2.3	18.8a,c±3.6
C18∶0 (µg/ml)	23.1±0.4	49.5^a^±6.2	38.8±6.4	28.2±1.3	24.2^c^±2.3	25.1±0.9
C16∶1 (µg/ml)	7.0±0.5	1.8^a^±0.3	3.8^a^±0.2	5.3±0.4	3.3^a^±0.5	2.0^a^±0.6
C18∶1 (µg/ml)	50.0±2.7	65.3±6.7	60.3±6.9	66.7±3.0	53.3±6.7	53.8±4.9
C18∶2 (µg/ml)	22.7±1.3	63.4^a^±5.7	52.2^a^±5.2	26.3±1.8	46.8^a^±3.5	38.7^a^±4.1
C20∶4 (µg/ml)	17.7±0.5	82.4a,b±6.1	35.5^a^±4.7	20.6±3.4	36.7^c^±2.3	22.6±4.1
C22∶6 (µg/ml)	4.7±0.2	26.9a,b±1.6	6.8±6.1	8.0±1.5	6.3^c^±0.75	5.4±0.4

The values presented are the means±SEM. For weight and caloric intake, *n* = 50 to 70, for plasma hormone and metabolite determinations, *n* = 10 to 15, for FA level determination, *n* = 7, and for Piximus analysis and organ weights, *n* = 5.

a *p*<0.05 for comparison with control,

b *p*<0.05 for comparison between OP and OR mice,

c *p*<0.05 for comparison between the F1LM and F2OM mice, assessed by Kruskal-Wallis tests followed by Dunn’s *post hoc* tests.

Determinations of several metabolites and hormone parameters (Table 1) showed that obesity was associated with hyperleptinemia and that the development of T2D was associated with higher plasma glucose and insulin concentrations in OP mice. In a previous study [21], we performed oral glucose tolerance tests (OGTT) and intraperitoneal insulin tolerance tests (ITT): OR2 females and CD-fed normal control mice displayed similar responses to insulin. Glucose homeostasis was impaired by high-fat feeding in OP1 and OR1 mice, and in OP2 mice. OP1 mice were hyperglycemic and hyperinsulinemic, whereas OP2 mice were hyperinsulinemic but not hyperglycemic, and OR2 mice were normoglycemic and normoinsulinemic at 24 weeks. Glycemia and insulinemia were thus similar in lean OR2 females and control mice (CD1 and CD2). OR1 and OR2 mice remained normoglycemic and normoinsulinemic. HOMA index values indicated abnormal fasting glucose concentrations in OP1 and, to a lesser extent, OP2 mice (table 1). All mice fed the HFD presented dyslipidemia, with high cholesterol and HDL-C levels, although this pattern was less pronounced in OR1 and OR2 mice.

The higher total circulating fatty acid concentration observed in the F1LM offspring (in both OP1 and OR1 mice) was not observed in the F2OM offspring (Table 1 and Figure 1). Detailed plasma fatty acid profiling showed that OP1 and OP2 mice had lower levels of C16∶1 and higher levels of polyunsaturated n-6 C18∶2 and C20∶4 than their respective controls. OP1 mice displayed specific modifications, with higher levels of C18∶0 and C22∶6, which were not found in the F2OM offspring (OP2 mice). Moreover, the changes in C18∶2 and C20∶4 FA levels were more marked in OP1 than in OP2 mice. OR1 and OR2 mice had lower levels of C16∶1 and higher levels of C18∶2 than CD1 and CD2 mice. These changes were of a similar magnitude to those observed in OP1 and OP2 mice. OR1 and OR2 mice had lower C16∶0 levels than control mice, this effect being particularly pronounced in OR2 mice. Higher levels of C20∶4 were found specifically in OR1 mice; but not in OR2 mice, which had C20∶4 levels similar to those of CD2 mice. C18∶1 levels were similar in all groups (Table 1).

Several studies have shown that maternal fat intake during the periconceptional/gestation/lactation period contributes to progression to non alcoholic fatty liver disease (NAFLD) in the offspring during adulthood [30], [31]. We show here that feeding a CD to obese, diabetic females during the periconceptional/gestation/lactation period limits the progression of HFD-induced NAFLD in the offspring. This conclusion is supported by the normalization of liver weight in the offspring born to obese mothers (F2OM), in both OP2 and OR2 females, and by the lower levels of steatosis revealed by hematoxylin and red oil staining (Wu *et al*. unpublished data), associated with clear changes in hepatic lipid metabolism, as reflected by adaptive changes in circulating fatty acid levels.

### Transcriptomic Data

**Figure 2:**
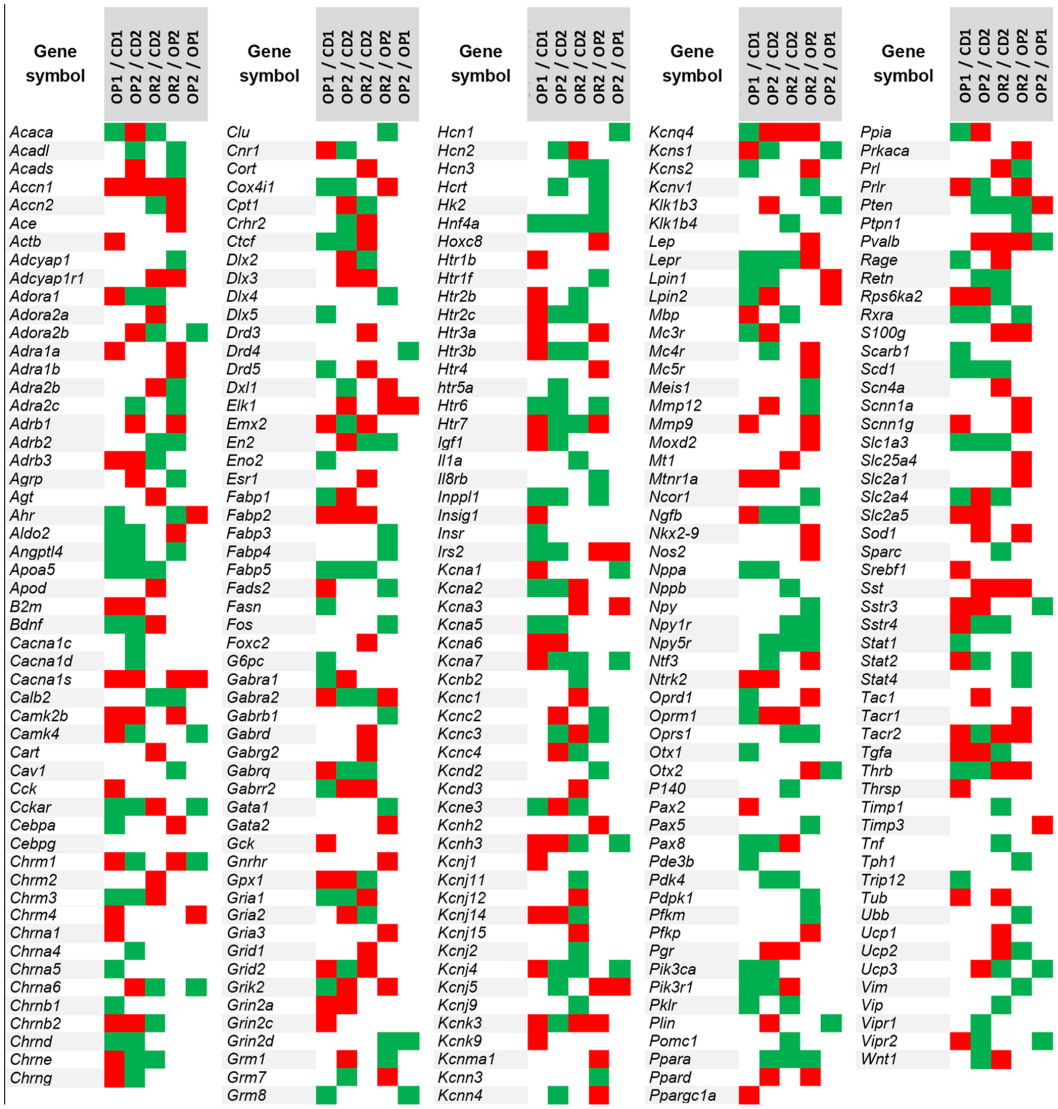
Heat map construction representing the differential expression of genes involved in metabolic function and neurotransmission. The heat map shows changes in the hepatic expression of genes encoding proteins involved in neurotransmission and genes encoding energy homeostasis-related proteins. Red, green and white squares represent upregulated, downregulated and unmodified genes, respectively.

**Figure 3:**
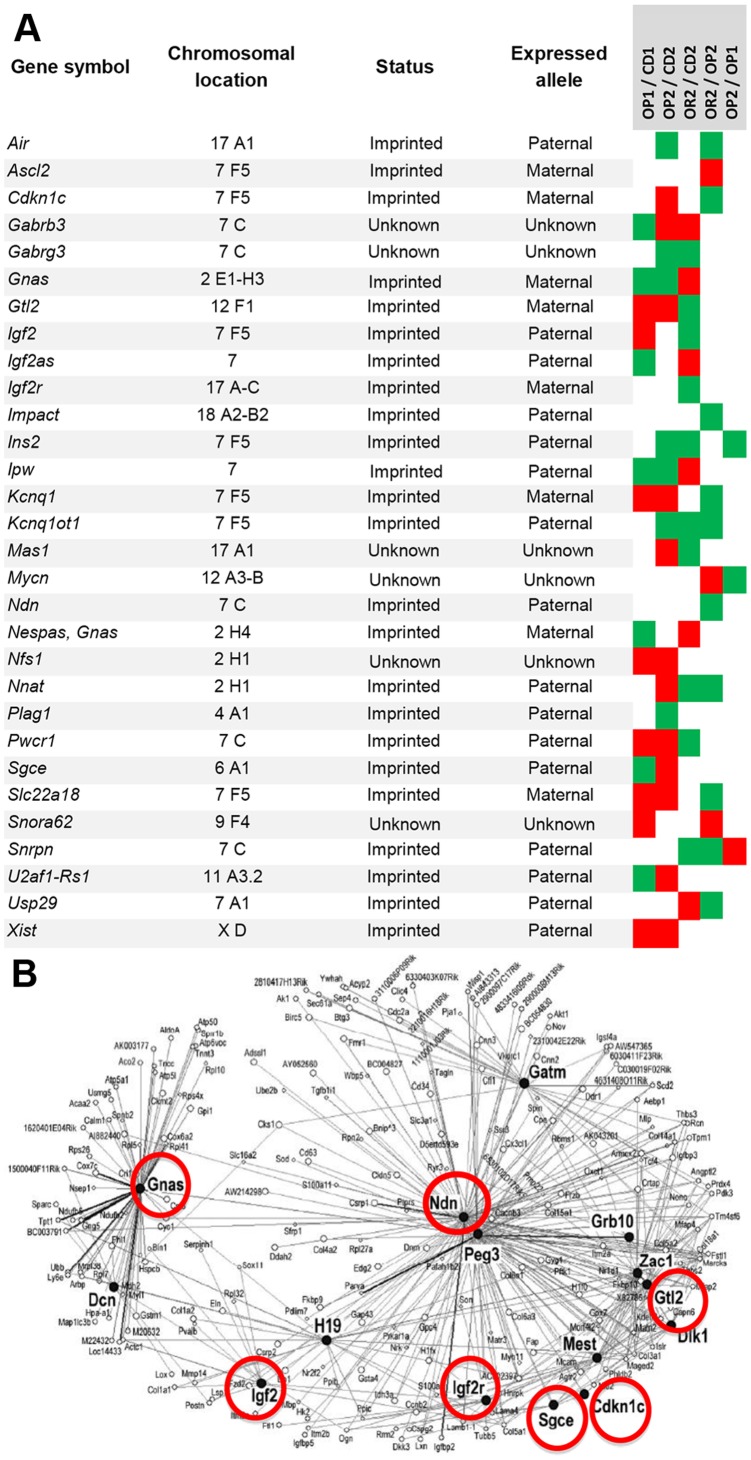
Heat map construction representing differentially expressed imprinted genes. (A) The heat map shows the changes in expression of imprinted genes in the liver in response to the HFD (OP1/CD1, OP2/CD2, OR2/CD2), with the trait of susceptibility/resistance to the obesogenic effects of HFD (OR2/OP2; indirect comparisons OR2/CD2 and OP2/CD2), or with a maternal effect (lean versus obese mother) (OP1/OP2); indirect comparisons (OP1/CD1 and OP2/CD2). Red, green and white squares represent upregulated, downregulated and unmodified genes, respectively. The status of the imprinted gene and the preferred parental allele for gene expression presented in this table at this time may subsequently be modified, as knowledge in this domain increases. Updates accessible via www.geneimprint.com). (B) Representation of the IGN network as described by Varrault *et* *al.* [50]. Red circles indicate the genes modulated in OR2 mice in response to the HFD and associated with maintenance of the lean phenotype (comparisons OR2/CD2 and OR2/OP2).

The microarray used contained 992 gene-specific 50-mer oligonucleotides generated from 63 imprinted genes (See Table S1), 256 genes encoding proteins involved in neurotransmission and 157 genes encoding proteins related to energy homeostasis [23]. A direct, dye-swapping comparison strategy was used to compare conditions, and dye-swaps were replicated several times, with at least four animals/group. Heat maps were constructed by compiling the differentially expressed genes (DEG) in the various comparisons (OP1/CD1, OP2/CD2, OR2/CD2, OR2/OP2, OP2/OP1), facilitating further indirect comparison between groups. The heat maps shown in Figures 2 and 3 highlight the dysregulated expression of three categories of genes: genes relating to metabolism, neurotransmission and imprinted genes.

The microarray results were confirmed by RT-qPCR, by determining the expression levels of 15 genes (listed in the legend to Figure S1) that appeared to be differentially expressed in at least one comparison between the various mouse groups (CD1, OP1, CD2, OP2, OR2; *n* = 5 per group). Expression values were normalized with the Genorm factor, and RT-qPCR expression ratios (OP1/CD1, OP2/N2, OR2/CD2, OR2/OP2 and OP2/OP1) were calculated and plotted against the corresponding microarray data (Figure S1). The RT-qPCR and microarray values were consistent for most of the 75 ratios, as indicated by the statistical significance of the linear regression equation (r^2^ = 0.718, *p*<10^−5^), highlighting the robustness of the microarray data.

As summarized in Table 2, we identified four different patterns of gene expression for the metabolic response to HFD, susceptibility/resistance and adaptation to the obesogenic HFD (Figures 2 and 3 and Table 2): 1) Resistance to the obesogenic diet: 260 genes were either specifically associated with the maintenance of a lean phenotype under a HFD in the OR2/CD2 comparison, distinguishing OR mice from controls (CD), or showed a striking difference in the OR2/OP2 comparison, distinguishing OR from OP mice. These genes were considered to be potentially involved in peripheral resistance to the obesogenic effects of the HFD*;* 2) Susceptibility to the obesogenic diet, with proneness to obesity: 287 genes associated with weight gain were either up- or downregulated in OP mice (comparisons OP1/CD1 and OP2/CD2). However, only 53 (19%) of these genes were dysregulated in a strictly similar manner by the HFD in both OP1 and OP2 mice, demonstrating differences in obesity phenotype despite similar weight gain; 3) Maternal effect: resistance or susceptibility to the obesogenic diet differed according to the leanness/obesity of the mother: 92 genes were differentially expressed between OP mice from the F1LM and F2OM groups. Thirty-five of these genes were identified by direct comparisons of OP2 and OP1 mice. Most of these genes (65%) were expressed less strongly in OP2 mice than in OP1 mice. In total, 57 genes were identified as differentially regulated between OP1 and OP2 mice (OP1/CD1 and OP2/CD2 comparisons) but similarly regulated between OP2 and OR2 mice (OP2/CD2 and OR2/CD2 comparisons). The pattern of gene expression in OP2 mice therefore prefigures the acquisition of resistance in OR2 mice. 4) Diet effect: 8 genes were either up- or downregulated by diet in both resistant and sensitive mice born to both lean and obese mothers (F1LM and F2OM) mice.

**Table 2:** Major functions involved in diet effect, proneness to obesity, resistance and adaptation to the obesogenic effects of a HFD.

	Obesity resistance specific	Obesity proneness specific	Maternal effect : lean versus obese	Diet effect
**Circulating fatty acids**				
	(−) C16∶0, in OR2 only	(−) C16∶1 n-7, in all; (+) C18∶2 n-6, C20∶4 n-6 in OP1, OP2, with OP1> OP2; (+) C20∶4 n-6, OP1<OR1<OP2 not OR2; (+) C18∶0, C22∶6 n-3, in OP1 only	(+) Total fatty acid, in OP1, OR1 only; (−) C16∶0, in OR2 only; (+) C18∶2 n-6, C20∶4 n-6, OP1> OP2; (+) C20∶4 n-6, OP1<OR1<OP2 not OR2	(−) C16∶1 n-7, in all; = C18∶1 n-3, in all
**Liver transcriptome Metabolism**				
Lipid uptake, storage and accumulation, metabolism, utilization	(−) *Acaca, Scd1, ApoA5, Cpt1, Ucp3, Pdk4, Fabp5, (by* Ppara) in OR2*;* (−) *Angptl4, Fabp4, Fabp3, Acadl, Cav1, Acads, Ucp2, Rxra, Fads2,* in OR2		(−) *Plin3, Timp3, Lpin1, Lpin2,* in OP with OP2>OP1	(−) *Apoa5, Fabp5, Hnf4a, Lepr, Scd1, Slc1a3;* (+) *Accn1, Fabp2* with OP>OR
Insulin signaling and sensitivity	(+/−) *Pten, Ptpn1, Inppl1, Pik3r1, Bdnf, Slc2a4 (Glut4),* in OR2 compared to OP2	(−) *Irs2, Pi3kr, Pi3kca, Hnf4a,* in OP1, OP2		
TG synthesis and lipid accumulation		(−) *Rxra, ApoA5, Angptl4, Scd1, Lpin1,* in OP1, OP2		
Lipogenesis		(+) *Fads2, Insig1, Ppargc1a, Srebf1, in OP1*	(+/−) *Fads2, Insig1, Ppargc1a, Srebf1,* (+) *in OP1* (−) *OP2, OR2*	
Lipid regulation and hepatic glucose production			(−) *Insr, Cebpa, G6pc, Fasn, Pde3b, Scarb1, Eno2,* in OP1 not in OP2, OR2	
Fatty acid oxidation			(+) *Acaca, Acads, Adrb1, Adrb3, Fabp2, Cpt1, Ppard, Ucp3,* in OP2 but not OP1	
Diverse			(−) *Retn, Npy5r, Ppara, Pdk4, Pten,* in OP2 and OR2 not in OP1	
**Liver transcriptome Neurotransmission**				
Calcium signaling			(−) *Adora2b, Caca1s, Chrm1, Camk4, CckAr, CckBr,* in OP1 to OP2 transition	
Thyroid signaling	(+/−)(+) *Thrb, Ucp1, Pax8, Ucp3, Adrbr3*, in OR2	(+/−) (−) *Thrb,* in OP		
K+ channels:	(+/−)(−) *Kcna2, Kcna3, Kcnd2, Kcnd3, Kcnj11,* in OR2	(+/−) (+) *Kcna2, Kcna5, Kcna6, Kcnh3, Kcnj14,* in OP	(−) *Kcna7, Kcnh3, Kcnj4, Kcns1, Kcna1, Hcn1,* in OP1 to OP2 transition; (+) *Kcna7, Kcnh9, Kcnj4, Kcnj1, Kcna1* in OP and (−) or ( = ) in OP2 and OR2	
Serotonin receptors	(+/−)(−) *Htr2c, Htr2b, Htr3b, Htr7*, in OR2		(+/−)(+) *Htr3a, Htr3b, Htr7, Htr1b*, in OP1 and (−) or ( = ) in OP2 and OR2	
Cholinergic/Nicotinic Receptors	(−) *Chrb2, Chrne, Chrna6,* in OR2; *+/−) Chrm3,* (+) in OR compared with OP; (−) (+) *Chrm3, Chrnb2, Chrnd*	(+/−) (+) *Chrm3, Chrnb2, Chrnd,* in OP1, OP2*;* (+) *Chrna1, Chrm4, Chrne*, in OP1 and (+) or = in OP2, OR2	(+) *Chrna1, Chrm4, Chrne,* in OP1 and (−) or ( = ) in OP2, OR2	
Orexigenic	(+/−) (−) *Npy, AgRP, Hcrt, Npy5r, Npy1r* in OR	(+/−) (+) *Npy, AgRP, Hcrt, Npy5r, Npy1r* in OP		
Anorexigenic	(+) *Mc4r, Mc5r, Sst,* in OR2 compared to OP2	(+/−) (−) *Mc4r, Mc5r, Sst,* in OP2 compared to OR2		
GABA receptors :	(+) *Gabra2, Gabrb3, Gabrd, Gabrg2, Gabrg3, Gabrq, Gabrr2,* in OR2			
**Liver transcriptome imprinted genes**				
Imprinted genes from different clusters:	(+/−) *Gnas, Gtl2, Mas1, Nnat,* between OP2, OR2*;* (-) *Igf2r, Gtl2* in 0R2	(+/−) *Gnas, Gtl2, Mas1, Nnat between OP2, OR2*; (+) *Slc22a18, Xist, Nfs1, Gtl2,* (−) *Gnas-Gsa*, in OP1, OP2	(−) *Ins2, Kcnq1ot, Gabrg3* in OP2, OR2 not in OP1	
PWS cluster	(+/−) *Ipw, Pwcr1,* between OP2 and OR2*;* (+) (+) *Snrpn, Gabrb3, Gabrg3,* in OR2	(+/−) *Ipw, Pwcr1,* in 0P1, OP2 compared to OR2	(−) *Gabrg3,* in OP2, OR2 not in OP1	
BWS cluster	(+) *Ascl2, Igf2-as*; (−) *Slc22a18, Kcnqt1, Kcnqt1ot, Cdkn1c, Igf2, Ins2, Cdkn1* in OR2	(+) *Kcnq1* in OP1, OP2,	(−) *Ins2, Kcnq1ot,* in OP2, OR2 not in OP1	
**Adipose tissue Gene expression**				
Adipogenesis, lipid storage Adipocyte size hypertrophy/hyperplasia	(−) *Lep, Peg1,* in OP2, OR2	(+) *Lep, Peg1,* in OP1	( = ) normalization of adipocyte size, in OP2 compared to OP1	
Energy expenditure		(+/−) *Adrb3* in OP1 and OP2 compared to OR2		(−) *Ucp1,*
Glucose utilisation		(+/−) *Pdk4* (−) in OP1, (+) in OR1, OP2, OR2	(+/−) *Pdk4* (+) in OP1, (+) in OR1, OP2, OR2	(−) *Glut4, Pparg*
Lipogenesis				(−) *Scd1*
Lipolysis				(−) *Acc1*
**Liver epigenetic marks**				
Global DNA methylation		(−) *in OP1 only*	(−) *in OP1 only*	
Histone K-acetylation				
Histone methylation				(+) *H3K4me3,* in OP2, OR2
**Liver epigenetic machinery**				
DNA methylation			(+) *Dnmt2,* in OP2,OR2 only	
Histone K-acetylation		(+) *Hat1*, in OP2 only		
Histone K-deacetylation	(+) *Hdac5,* in OR1 only		(+) *Hdac5,* in OR1	
Histone methylation	(−) *H3K9ac,* in OR2	(+) *Suv39h1, Suv39h2*, in OP1 only	(+) *Suv39h1, Suv39h2,* in OP1 only	(−) *Set7/9*
Histone K-demethylation	(−) *H3K9me1,* in OR2		(−) *Jhdm2a,* in OP2, OR2	
**Muscle epigenetic marks**				
Global DNA methylation		(−) in OP2 only	(−) in OP2 only	
**Muscle epigenetic machinery**				
DNA methylation	(+/−) *Dnmt2* in F2, (+) in OR2, (+) in OP2/CD		(+/−) (+) *Dnmt2,* in OP1, OR1, OR2, (+/−) (+) in OP2	
Histone K- acetylation		(+) *Hat1,* in OP2 only	(+) *Hat1*, in OP2 only, (+) *Gcn5,* in OP1, OR1 only	(+) *Gcn5,* in OP1, OR1 only
Histone K-deacetylation	(+) *Hdac5,* in OR1 only		(+) *Hdac5,* in OR1 only	
Histone K-methylation	(+) *Suv39h1,* in OP1, OR1, OR2 only; (+) *Suv39h2,* in OR1, OR2 only			
Histone K-demethylation			(−) *Jhdm2a,* in OP2, OR2, OP2<OP2	

(+/−) mirror-image pattern of gene expression; (−) decrease in gene expression; (+) increase in gene expression; ±dysregulated gene expression (either up- or downregulation).

**Expression of liver metabolism genes:** As shown in table 2, in OR2 mice, despite the higher lipid content of the HFD than of the CD, whole-lipid metabolism in the liver appeared to be restricted, with the strong repression of many genes involved in lipid storage and utilization, consistent with the normalization of liver weight and caloric intake. Genes regulated by Ppara and genes regulating lipid uptake, storage and utilization were repressed in OR2 mice. Several regulators of insulin signaling and sensitivity displayed differential expression between OR2 and OP2 mice. This suggests that the hepatic insulin signaling pathway may be better preserved in OR2 mice.

We clearly identified thyroid hormone signaling, which was upregulated only in OR2 mice, as a major pathway contributing to adaptation and, thus, to resistance to the obesogenic effect of the HFD. Thus, genes involved in lipid metabolism and regulated by Ppara were repressed and Thrb signaling was upregulated in obesity-resistant, but not in obesity-prone mice (Table 2).

Similar transcriptional responses, resulting in the well known abnormalities of T2D, such as changes to the expression of insulin signaling-related genes and lipid metabolism-related genes and features of hypothyroidism were observed in OP1 and OP2 mice mice born to both lean and obese mothers (Figure 2).

However, OP mice also displayed specific expression patterns potentially responsible for the differences in metabolic phenotype observed between mice born to either lean or obese mothers. Specifically in OP1 mice, genes linked to *de novo* lipogenesis were activated, despite the lipid overload induced by HFD. This may have led to the deposition of larger amounts of lipids and to the development of steatosis in these mice. The expression of several genes involved in lipid regulation and hepatic glucose production was also considerably modified in OP1 mice, whereas no such changes were observed in OP2 and OR2 mice. Genes involved in lipid storage and accumulation were more strongly repressed in OP2 mice than in OP1 mice. In addition, OP2 mice presented a striking upregulation of genes involved in fatty acid oxidation that was not observed in OP1 mice, based on the OP1/CD1 comparison. This may reflect an increase in the capacity to process excess fatty acids, preventing OP2 mice from developing hepatic steatosis (Table 2). Thus, in F1LM and F2OM mice, specific metabolic pathways were progressively recruited to spare the liver from lipid accumulation and hypertrophy and to maintain its capacity to respond to insulin.

Diet sensitivity was noted for genes or marks dysregulated in the same way in all groups fed the HFD. Transcriptomic analysis of the liver showed that eight lipid metabolism genes were similarly upregulated or downregulated in all groups fed the HFD, with OP mice generally more strongly affected than OR mice (Table 2).

**Expression of liver neurotransmission genes:** The traits of proneness or resistance to the obesogenic effects of a HFD were associated with major changes in the transcriptional regulation of neurotransmission genes in the liver. The central modulation of the expression of these genes to control food intake and energy expenditure is well documented, but it is intriguing that some of these “neurotransmission” genes appear to be modulated in the same way in the liver and the central nervous system.

Many genes encoding GABAergic receptors were dysregulated in OR2 mice, suggesting a potential GABAergic control of metabolism, as reported in studies on hepatic glucose production [32] (Table 2).

Nicotinic acid receptors were downregulated in OR mice, whereas *Chrm3* displayed the opposite pattern, being upregulated in OR2 mice. This is of particular interest, because acetylcholine (Ach) signaling has been implicated in the regulation of hepatic glucose metabolism and insulin responsiveness [33], [34] and the effects of Ach on glycogen synthesis and neoglucogenesis seem to be mediated by this receptor [35]. (Table 2).

In addition, OR2 mice had lower levels of orexigenic gene expression than OP2 mice, and displayed an upregulation of genes encoding anorexigenic factors in response to positive energy balance (OP/OR). A similar pattern has been reported for the hypothalamus, but this is the first report of such a pattern for the liver. In the context of Npy signaling, the specific downregulation of both *Npy5r* and *Npy1r* in OR2 mice is important, because these two genes underlie the potent effect of Npy on both feeding regulation and the control of energy expenditure [36], [37]. (Table 2).

The development of obesity and T2D in mice born to both lean and obese mothers was associated with the dysregulation of many genes encoding potassium channels and several genes encoding cholinergic receptors (Figure 2), and the expression of some of these genes was modified in the opposite manner in OR mice (Table 2).

Many genes encoding potassium channels, cholinergic and serotoninergic receptors were upregulated in OP1 mice but repressed or unaffected in OP2 and OR2 mice. (Table 2).

The transition from an OP1 to an OP2 phenotype (OP2/OP1 comparison) was also associated with a global repression of calcium signaling-related genes and of genes encoding K^+^ (potassium) channels. (Table 2).As recently revealed by genome-wide surveys of gene expression in 15 different tissues and cell lines, up to 94% of human genes generate more than one product. This suggests that our knowledge of the tissue-specific functions of genes is far from complete and that caution is required in interpreting the dysregulation of genes in a given tissue [38], [39]. Further studies are therefore required to determine the functional significance of these results, but these changes in the expression of genes involved in peripheral neurotransmission may play as important a role as changes in the expression of classical metabolic genes in the response to a HFD and resistance/susceptibility to its obesogenic effects and, thus, to obesity. Nevertheless, crosstalk between centrally (hypothalamus) and peripherally (liver) modulated gene networks may result in concerted effects on the control of food intake and energy expenditure (Table 2).

**Expression of imprinted genes in the liver:** Although not yet formally demonstrated, it has been suggested that genomic imprinting may act as a buffering system or “rheostat”, supporting adaptation to changes in environmental conditions by silencing or increasing the expression of monoallelically expressed genes [36], [40], [41], [42]. As indicated above, the precise roles of most of these genes in the liver and in adulthood remain to be determined, but a close relationship with developmental programming processes has been demonstrated [38], [39](Table 2).

OR2 mice born to obese mothers displayed changes in the expression of several imprinted genes from the 7F5 cluster *Igf2/H19,* with the upregulation of *Igf2as*, but the repression of *Ins2* and *Igf2* (Figure 3). Thirteen imprinted genes were differentially expressed between OP2 and OR2 mice, including genes from the 7F5 cluster implicated in Beckwith-Wiedemann Syndrome (BWS), with the upregulation of *Ascl2* and *Igf2as* and the repression of 7 genes including *Igf2* (Figure 3) in OR2 mice, and six imprinted genes displaying strict mirror-image patterns of expression in OR2 and OP2 mice (Table 2).

In the OR2/CD2 and OR2/OP2 comparisons, several genes from the Prader-Willi syndrome (PWS) region also displayed modified expression in OR2 mice, both in response to the HFD and in association with the maintenance of a lean phenotype (Table 2).

The expression of three imprinted genes was unaffected in OP1 mice, but repressed in both OP2 and OR2 mice, consistent with a role of these genes in adaptation to changes in environmental conditions (Table 2).

Several imprinted genes were found to be upregulated in OP mice born to both lean and obese mothers, whereas others including *Gnas-Gsa* and *Ipw* were repressed (Table 2).

Thus, a large proportion of imprinted genes were dysregulated in mice fed a HFD, clearly following the adaptive response to the obesogenic diet. Several genes, such as *Igf2/Igf2as* and *Igf2r,* play a role in embryonic and placental growth, by governing nutrient availability. In addition to *Cdkn1c*, *Kcnqt1* and *Kcnqt1ot*, these genes are involved in the overgrowth observed in BWS [43]. The *Meg3/Gtl2* gene plays important roles in postnatal growth, adaptation and adiposity. The *Nnat* gene is involved in regulating insulin secretion in pancreatic beta cells and is dysregulated in models of litter size-associated overnutrition [44], [45]. Other genes, such as *Snrpn, Ipw, Pwcr1*, *Gabrb3* and *Gabrg3,* have been identified as contributing to obesity and other symptoms of PWS. Of particular interest, *Gnas-Gsa,* which displayed opposite patterns of regulation in OP and OR mice, is one of the multiple products of the *Gnas* locus, which modulates susceptibility to obesity through various effects on insulin sensitivity, glucose and lipid metabolism [46], [47], [48]. (Table 2).

Several dysregulated imprinted genes (*Gnas*, *Igf2*, *Igf2r*, *Gtl2*, *Cdkn1* and *Sgce*) constitute important nodes in the recently described network of coregulated imprinted genes (IGN) [49], [50]. With the exception of *Sgce,* all are modulated under a HFD in OR2 mice and associated with the maintenance of leanness (Figure 3). The mRNA levels of most of the genes of the IGN have been shown to be significantly altered, in a coordinated fashion, in placentas obtained after *in vitro* fertilization and undergoing apparently normal development. This global modulation of imprinted genes is thought to act as a compensatory process, correcting the potential dysfunction of the placenta [51]. We recently demonstrated that a maternal HFD during pregnancy also results in the deregulation, in the placenta, of clusters of imprinted genes controlling many cellular, metabolic and physiological functions potentially involved in adaptation and/or evolution [26]. The global dysregulation of genes from the IGN in the livers of OR mice could therefore also be interpreted as an adaptive process triggered to preserve liver function and necessary, under deleterious conditions of lipid overload, to maintain a fairly normal phenotype (Table 2).

**Figure 4:**
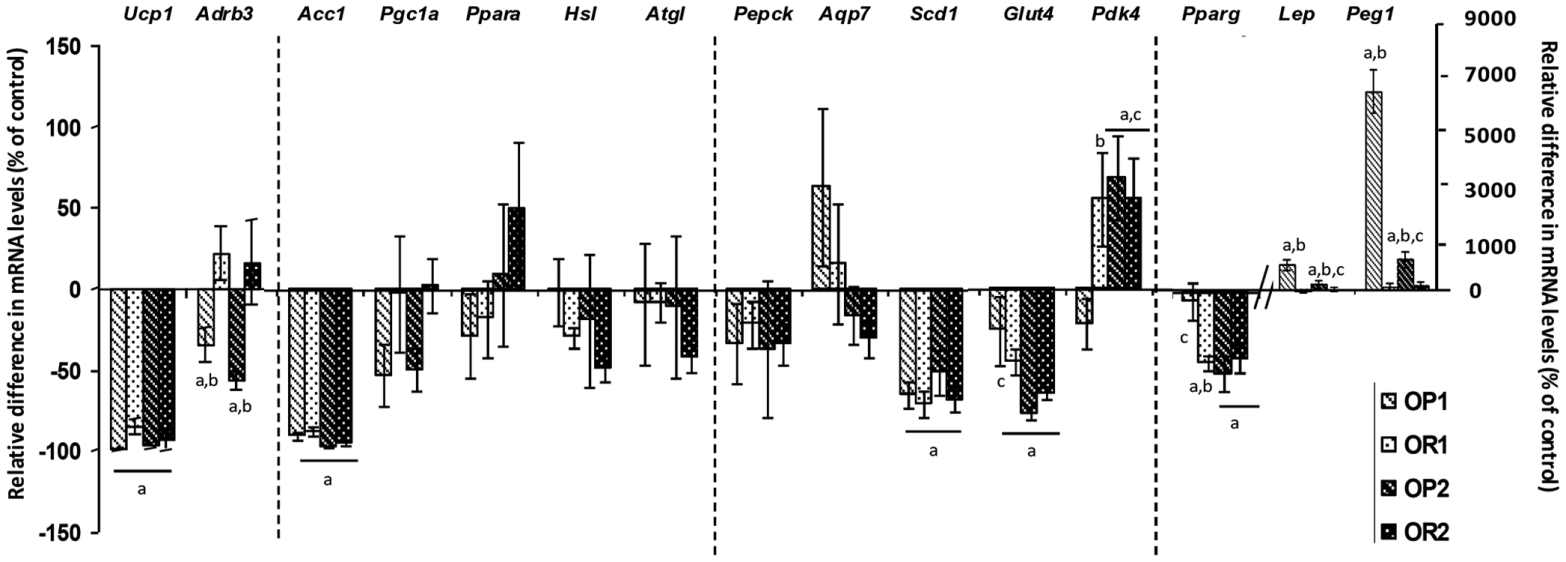
Analysis of mRNA levels for key adipogenic genes by RT-qPCR on the adipose tissue of mice fed either the CD or the HFD, born to either lean or obese/diabetic mothers (F1LM and F2OM). The values shown are the ratios between OP1 or OR1 and CD1 and between OP2 or OR2 and CD2 (Figures 9 and 10). They are expressed as the mean±SEM, *n* = 6 per group. ^a^ *p*<0.05 for comparison with control CD, ^b^ *p*<0.05 for comparison between OP and OR mice, ^c^ *p*<0.05 for comparison between F1LM and F2OM, assessed by Kruskal-Wallis tests followed by *post hoc* Dunn’s tests.

**Adipose tissue gene expression:** As shown in figure 4, levels of gene expression in the adipose tissue were determined as a ratio between OP1 or OR1 and CD1 and between OP2 or OR2 and CD2 mice. The levels of expression (as assessed by RT-qPCR) of key genes relating to adipogenesis and adipocyte size (*Pparg*, *Lep*, *Peg1*), energy expenditure (*Ucp1*, *Adrb3*), glucose utilization (*Slc2a4(Glut4)*, *Pdk4*), lipogenesis (*Scd1*, *Aqp7*, *Pepck*) and lipolysis (*Hsl*, *Atgl*, *Acc1*, *Ppara* and *Pgc1a*) were modified by resistance or susceptibility to obesity, or by maternal effects (lean versus obese mother) or by diet (Figure 4 and Table 2).

*Adrb3*, *Lep* and *Peg1* were dysregulated in OP1 and OP2 mice only, and were therefore strictly associated with proneness to obesity. *Ucp1*, *Acc1* and *Scd1* were similarly downregulated in response to the HFD in OR and OP mice born to both lean and obese mothers, suggesting an effect of diet (Table 2). Finally, the expression of *Pepck*, *Aqp7*, *Atgl*, *Hsl*, *Ppara* and *Pgc1a* did not differ between conditions.

**Figure 5:**
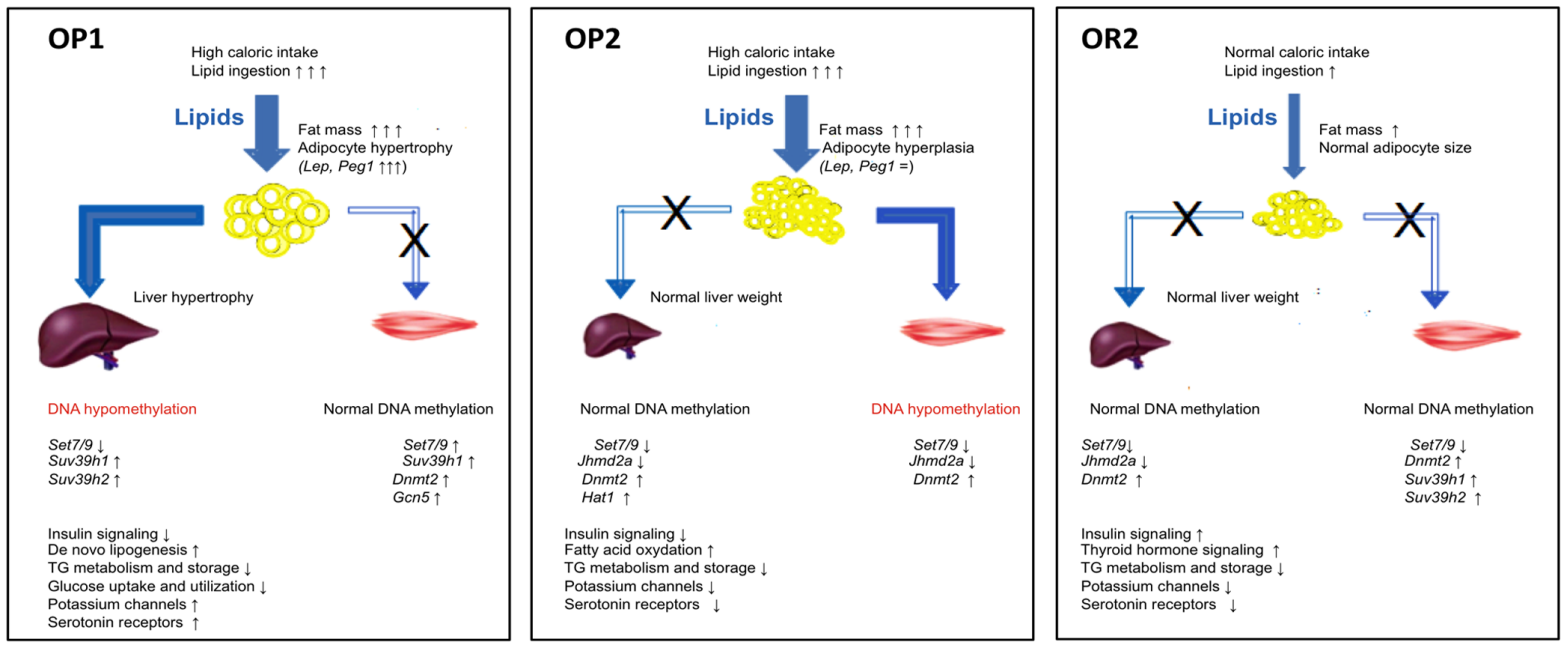
Schematic diagram summarizing the transcriptional data obtained for the liver, muscle and adipose tissue and the results of epigenetic studies in OP1, OP2 and OR2 mice. Blue arrows represent potential lipid fluxes. In the hyperphagic OP1 and OP2 mice, lipid ingestion was much greater than in OR2 mice, in which caloric intake was normalized. In OP mice, excess lipids were initially stored in the adipose tissue, leading to adipocyte hypertrophy in OP1 mice and hyperplasia in OP2 mice, as a function of the level of expression of *Lep* and *Peg1*. In OR2 mice, lipids were stored in the adipose tissue without adipocyte abnormalities or ectopic storage, as in mice supplied with limited amounts of lipid. In OP1 mice, the excess lipids were stored in the liver, contributing to hepatic hypertrophy. Transcriptomic data indicated that *de novo* lipogenesis was activated in OP1 mice and that insulin signaling was greatly disturbed. In OP2 mice, genes related to insulin signaling were less affected, whereas genes involved in fatty acid oxidation were globally upregulated. Changes to hepatic metabolism, together with the probable redirection of lipids to muscle thus spared the liver from lipid accumulation. Finally, in OR2 mice, lipid metabolism as a whole was downregulated, whereas thyroid hormone signaling was upregulated. The HFD response was also associated, in OP1 mice, with an upregulation of potassium channels and serotonin receptors, subsequently reversed in both OP2 and OR2 mice. Changes in DNA methylation were observed in the livers of OP1 mice and the muscle of OP2 mice. In the liver, *Set7/9* expression was decreased by the HFD in mice born to either lean or obese/diabetic mothers (F1LM and F2OM), whether OP or OR. In the livers of OP1 mice, DNA hypomethylation was associated with an upregulation of *Suv39h1* and *Suv39h2* expression, whereas, in both OR2 and OP2 mice, normal DNA methylation was associated with a decrease in *Jhdm2a* expression and an increase in the level of *Dnmt2* mRNA. In muscle, normal DNA methylation was associated with an upregulation of *Suv39h1, Set7/9* and *Dnmt2* in OP1 and OR2 mice, contrasting with the lower level of expression of the *Set7/9* and *Dnmt2* genes in the muscle of OP2 mice presenting DNA hypomethylation.

Changes in the adipose tissue also supported the metabolic adaptation of the mice according to maternal leanness or obesity status. *Pparg*, *Glut4* and *Pdk4* were differentially expressed in OP mice, with levels in OP2 mice similar to those in OR1 and OR2 mice. Levels of *Pparg* and *Glut4* expression were lower and levels of *Pdk4* expression were higher in OR1, OR2 and OP2 mice than in CD mice, whereas the expression of these genes was unaffected in OP1 mice. This response switches the energy source from glucose to fatty acids, to maintain blood glucose levels (Figure 5) [52]. In OP1 mice, lipid storage was associated with increases in the expression of both *Lep* and *Peg1,* reflecting adipocyte hypertrophy, which could lead to a worsening of the metabolic phenotype by promoting the development of a pro-inflammatory state and insulin resistance [37]. By contrast, OP2 mice, despite displaying a weight gain similar to that of OP1 mice, had much lower levels of *Lep* and *Peg1* expression, suggesting a normalization of adipocyte size and an expansion of fat mass through hyperplasia, a major feature of the adaptive response [53], [54]. Thus, adipose tissue displayed a pronounced dysregulation of gene expression, with an upregulation of genes involved in lipid storage and associated with adipocyte hypertrophy in OP1 mice, and hyperplasia in OP2 mice, according to maternal leanness or obesity status (Table 2).

### Major Functions and Networks of Interactions in Proneness, Resistance and Maternal Effects

Genes either up- or downregulated in OR1 and OR2 mice reflect proactive mechanisms, leading to resistance to the deleterious effects of a HFD, combining both peripheral mechanisms conferring metabolic adaptation to the relative disproportion of lipids and a probable central mechanism for preventing hyperphagia. We did not address the issue of hyperphagia directly in this study, but the dysregulation of neurotransmission genes in the liver strongly suggests that cross-talk occurs between the two mechanisms. However the mechanisms involved in OR1 and OR2 mice are strikingly different, suggesting that the stochastic resistance in the female offspring of lean mothers does not involve the same pathways. By contrast, the genes dysregulated in OP1 and OP2 obese mice probably reflect the pathological condition developed after 20 weeks on a HFD, leading, in different ways in OP1 and OP2 mice, to massive obesity and T2D. We investigated the functions and pathways most clearly related to the observed phenotypic characteristics, by carrying out Ingenuity pathway analysis (IPA) on the liver.

**Figure 6:**
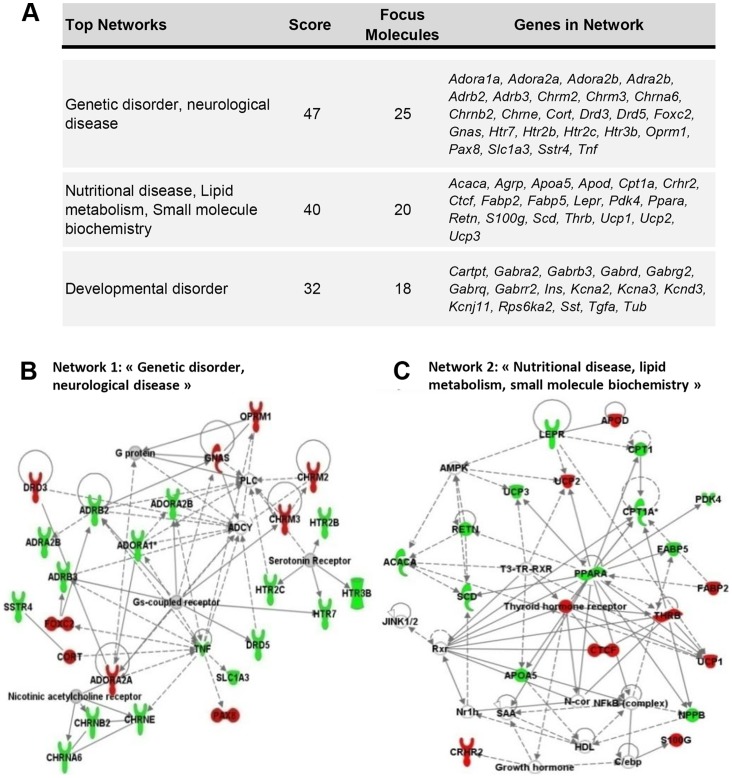
Resistance to the obesogenic effects of the HFD: Major networks identified by IPA analysis of the genes involved in the response and adaptation to HFD of OR2 mice. (A) These networks were built from the 142 genes differentially expressed between OR2 and CD2 mice (direct comparison OR2/CD2). Only the first two networks are represented in (B) and (C). The node color indicates the level of expression of the genes: red, upregulated; green, downregulated.

The response to a HFD in OR2 mice (OR2/CD2 comparison) was associated with changes in the expression of genes that appeared to be clustered into several networks (Figure 6A). The two main networks, “Genetic Disorders, Neurological Diseases” and “Nutritional Diseases, Lipid Metabolism and Small-Molecule Biochemistry”, are presented in Figure 6B and 6C. Network 1 highlights the global repression of serotonin receptors and an upregulation of Ach nicotinic acid receptors (Figure 6B). Network 2 highlights changes in the expression of genes directly involved in lipid metabolism (Figure 6C), with the repression of genes regulated by Ppara and the upregulation of Thrb signaling. Many genes encoding GABAergic receptors and potassium channels involved in neurotransmission were also dysregulated, and some clustered into a third network, “Developmental Disorders” (not shown).

The 128 genes differentially expressed between OR2 and OP2 mice were associated, in particular, with “nutritional and metabolic disease”. They included genes related to adiposity, dyslipidemia, T2D and organ damage, together with genes linked to steatohepatitis and inflammation (Table S2). The main biological function altered in obesity resistance was “Cell-to-Cell Signaling”, due to the dysregulation of many genes encoding receptors modulating G-protein signaling (adrenergic, glutamatergic and serotoninergic receptors), proteins modulating intracellular calcium levels and potassium channels. OR2 mice displayed lower levels of orexigenic gene expression than OP2 mice, and an upregulation of genes encoding anorexigenic factors (Table 2). Moreover, strict mirror-image patterns of expression were observed for 42 genes in OR2 and OP2 mice, in the OR2/CD2 and OP2/CD2 comparisons. These genes included 25 neurotransmission-related genes, many encoding potassium channels, six imprinted genes and several other genes involved in thyroid hormone signaling and insulin sensitivity.

The 53 genes dysregulated in both OP1 and OP2 mice had functions in pain; metabolic and nutritional disorders, with the altered genes related to adiposity, diabetes and hypothyroidism; obesity and hepatic system disorder, with genes associated with liver cancer and hematological disorders (Table S3A). Forty-five of these genes clustered into three main networks relating to “Lipid Metabolism, Molecular Transport, Small-Molecule Biochemistry”, “Endocrine System Development and Function” and “Organismal Injury and Abnormalities” (Table S3B).

**Figure 7:**
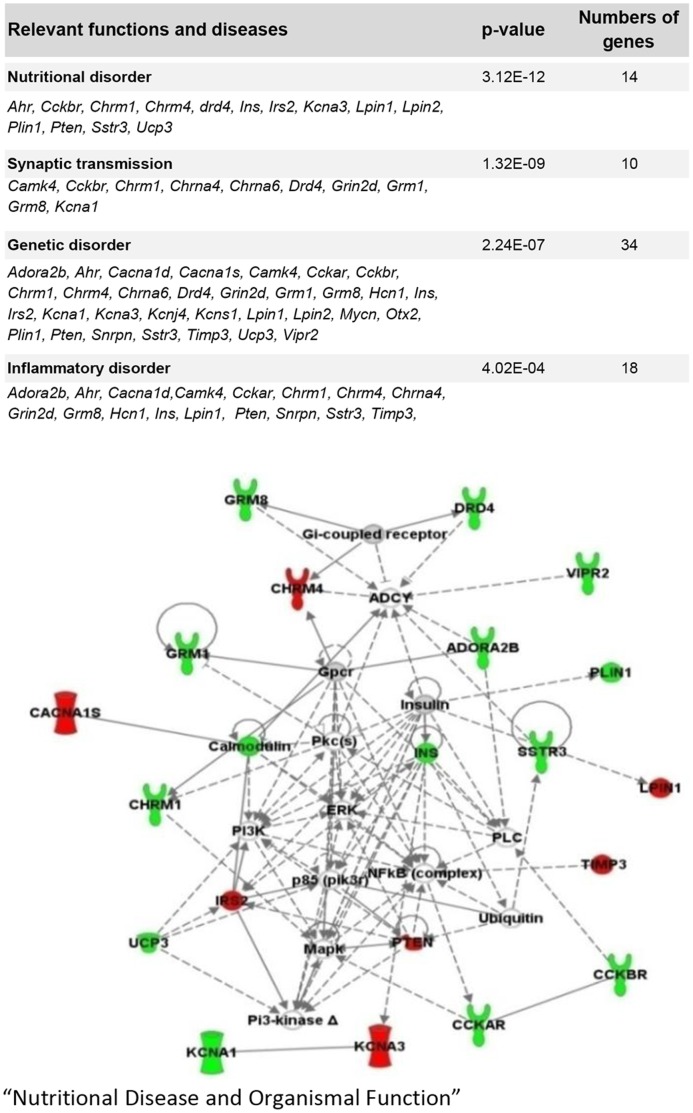
Maternal effect (lean versus obese mother): Relevant functions and diseases, and representation of the major network of interaction identified by IPA analysis of the genes differentially expressed between OP1 and OP2 obese mice. The 35 genes differentially expressed between the obese mice born to lean and obese/diabetic mothers (F1LM and F2OM) obtained in the direct OP2/OP1 comparison were subjected to IPA analysis. The network shown contains 20 focal genes with a score of 50. The node color indicates the expression levels of genes: red, upregulated; green, downregulated.

In total, 57 genes involved in the response to HFD were differentially regulated between OP1 and OP2 mice (comparisons between OP1/CD1 and OP2/CD2) but similarly regulated in OP2 and OR2 mice (comparisons between OP2/CD2 and OR2/CD2). Many of the genes upregulated in response to a HFD in OP1 mice and repressed or unmodified by a HFD in both OP2 and OR2 mice were related to neurotransmission, encoding potassium channels, cholinergic and serotoninergic receptors, or involved in lipogenesis. In the OP2/OP1 comparison, most (65%) of the 35 genes displaying differential regulation between F1LM and F2OM obese mice were repressed and related to the following functions and diseases: “Nutritional Disorder”, “Synaptic Transmission”, “Genetic Disorder” and “Inflammatory Disorder” (Figure 7). In OP2 mice, the genes globally repressed with respect to OP1 mice were involved in lipid storage and accumulation, related to the calcium signaling pathway, or encoded potassium channels. These genes clustered together in a network related to “Nutritional Disease and Organismal Function” (Figure 7). This maternal effect (obese versus lean mother) was characterized, in adipose tissue, by a mirror-image for the expression of *Pdk4*. There were also specific changes in the expression of several genes of the epigenetic machinery in both the liver and muscle (Table 2).

### Epigenetic Analyses

**Global DNA methylation and histone modifications:** Chromatin is regulated by modifications of about 80 known histones and DNA modifications, generating docking sites for enzymes that modify chromatin or remodel nucleosomes. DNA methylation is often associated with gene repression, whereas histone methylation may be associated with gene activation or gene repression, depending on the residue methylated, the degree of methylation and the position of the methylated residue with respect to the gene sequence. Histone acetylation in the promoter and coding regions of genes is essentially correlated with transcriptional activation [55]. Providing an additional level of complexity, recent studies have highlighted the crosstalk between marks [56]. However, given the large number of epigenetic marks and modifiers in existence, we still know very little about the ways in which the diverse environmental factors that interfere with different chromatin landscapes and players induce global chromatin modifications and control the expression of individual genes [57].

**Figure 8:**
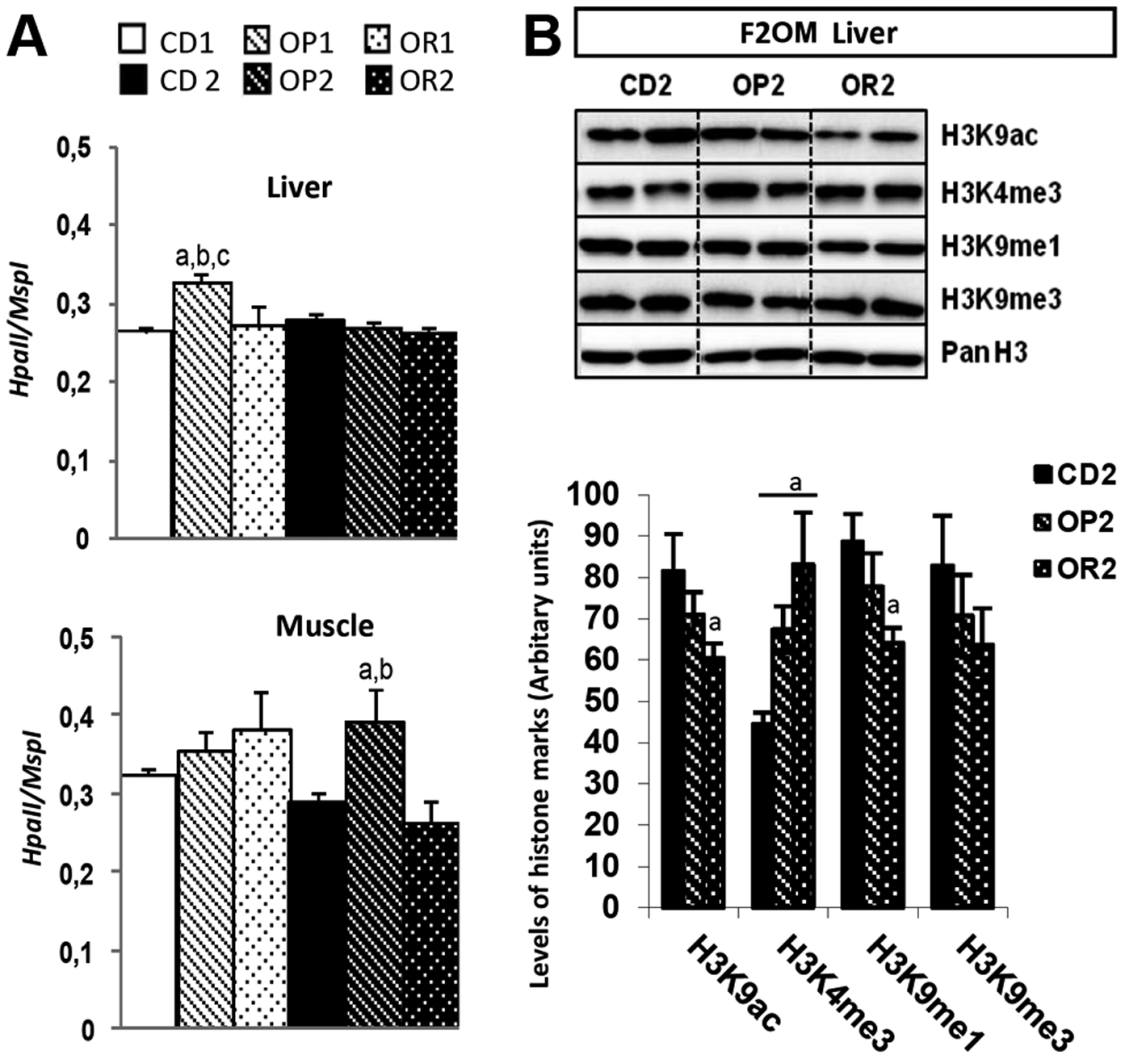
Analysis of global epigenetic modifications in several organs of mice fed either the CD or the HFD, born to either lean or obese/diabetic mothers (F1LM and F2OM). (A) Global DNA methylation analysis by LUMA in the liver and muscle of CD, OR and OP females, *Hpa*II/*Msp*I levels indicate the degree of CCGG unmethylation. (B) Global analysis of posttranslational histone modifications by western blotting, for the liver of F2OM females. The values shown are the mean±SEM. ^a^ *p*<0.05 for comparison with control CD, ^b^ *p*<0.05 for comparison between OP and OR mice, ^c^ *p*<0.05 for comparison between the F1LM and F2OM mice born to lean and obese mothers, respectively, assessed by Kruskal-Wallis tests and *post hoc* Dunn’s tests.

We first evaluated the global level of DNA methylation with the LUMA technique. DNA methylation levels were found to be lower than normal in the livers of OP1 mice only (Figure 8A), and in the muscles of OP2 mice only (Figure 8A). No change in DNA methylation was observed in the adipose tissue or kidneys (data not shown). The DNA hypomethylation observed in mice on a HFD was not due to a lower intake of methyl donors, as the amounts of these compounds (choline, betaine, biotin, folic acid, methionine) provided by the diet were adjusted according to caloric content. However, efficiency of use of these substrates and of the cellular pathways involved may differ between OP and OR mice, thus contributing to differences in DNA methylation levels [58], [59], [60](Table 2).

Similarly, global changes to histone acetylation and methylation may also be observed under diabetic stimuli, such as exposure to high glucose concentrations, with effects on key genes related to diabetes, glycemic memory and inflammation [61], [62], [63], [64]. The experiments described above used up large amounts of tissue. As a result, the remaining samples were sufficiently large for testing only for the OP2 and OR2 groups born to obese mothers (F2OM). Four posttranslational histone modifications, H3K9ac, H3K9me1, H3K9me3, and H3K4me3, were analyzed by western blotting in the livers of OP2, OR2 and CD2 mice (Figure 8B). Obesity resistance was characterized by a specific decrease in two histone marks – H3K9ac and H3K9me1 – in the livers of OR2 mice. Both H3K9 acetylation (H3K9ac) and H3K9me1 levels were significantly lower in OR2 mice than in CD2 mice, but did not differ significantly between OR2 and OP2 mice, suggesting an effect of diet. Levels of H3K4me3, a mark often associated with transcriptionally active chromatin, were significantly higher in both OP2 and OR2 mice, than in control mice, suggesting a diet effect. No differences were found in the levels of H3K9me3, a mark often associated with transcriptionally inactive chromatin (Table 2).

**Figure 9:**
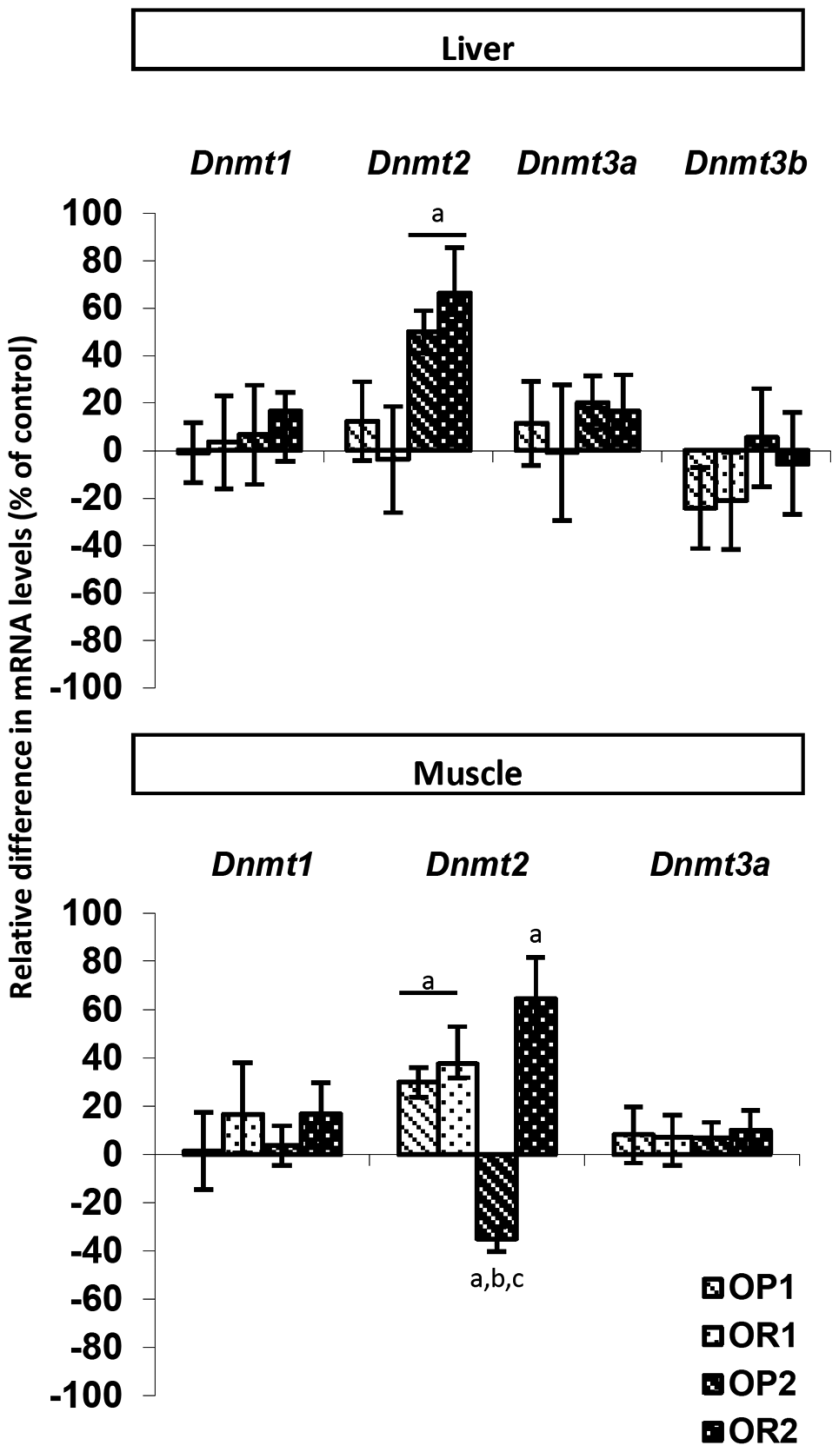
Analysis, by RT-qPCR, of the expression of genes encoding DNA methyltransferase enzymes in the liver and muscle of mice fed the HFD, born to either lean or obese/diabetic mothers (F1LM and F2OM). The values shown are the ratios between OP1 or OR1 and CD1 and between OP2 or OR2 and CD2. They are expressed as the mean±SEM, *n* = 8 per group. ^a^ *p*<0.05 for comparison with control CD, ^b^ *p*<0.05 for comparison between OP and OR mice, ^c^ *p*<0.05 for comparison between the F1LM and F2OM born to lean and obese/diabetic mothers, respectively, assessed by Kruskal-Wallis tests and *post hoc* Dunn’s tests.

**Figure 10:**
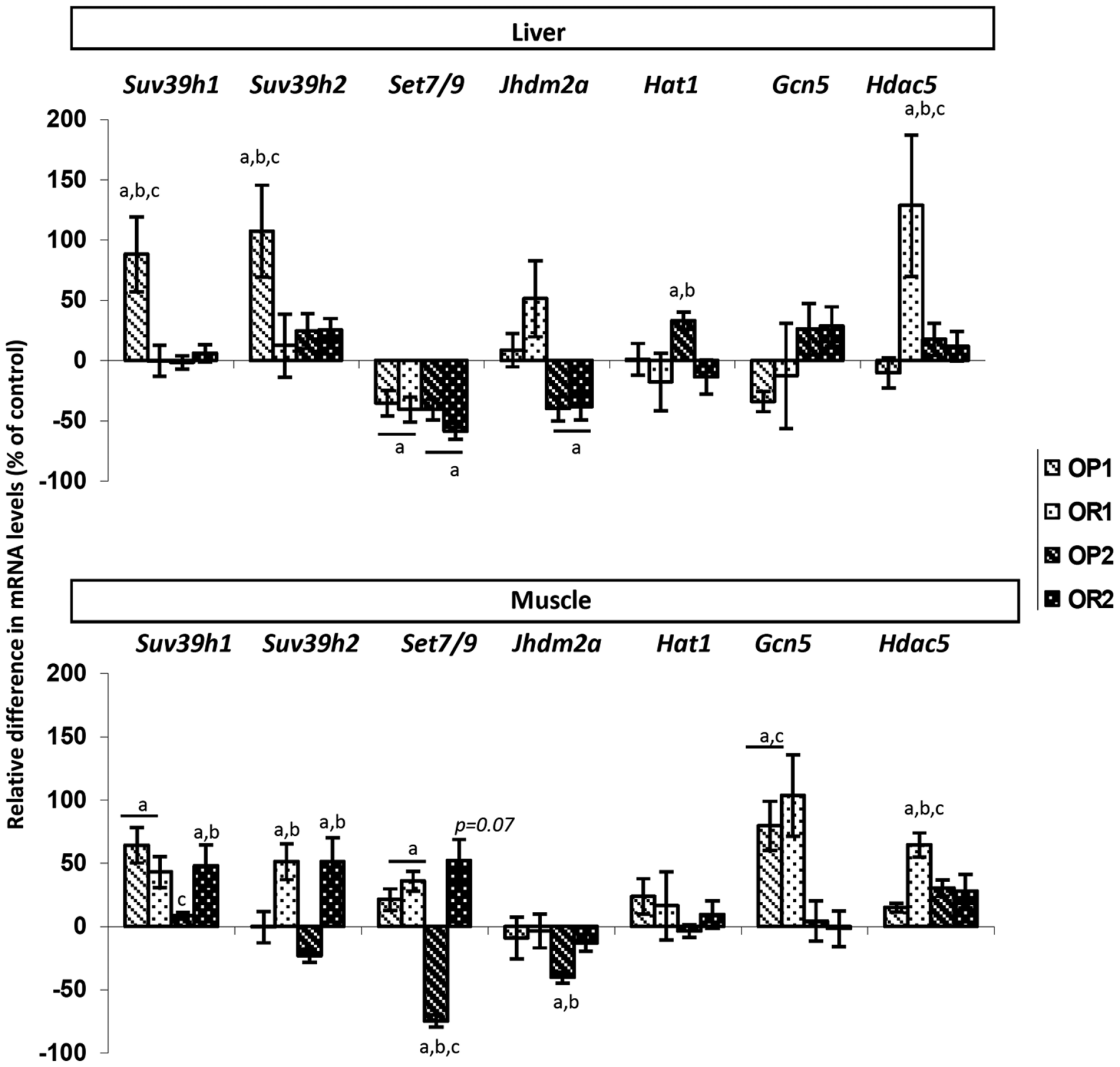
Analysis, by RT-qPCR, of the expression of genes encoding histone-modifying enzymes in the liver and muscle of mice fed the HFD, for both lean and obese mothers. The values shown are the ratios between OP1 or OR1 and CD1 and between OP2 or OR2 and CD2. They are expressed as the mean±SEM, *n* = 8 per group. ^a^ *p*<0.05 for comparison with control CD, ^b^ *p*<0.05 for comparison between OP and OR mice, ^c^ *p*<0.05 for comparison between the F1LM and F2OM (maternal effect: lean versus obese mother), assessed by Kruskal-Wallis tests and *post hoc* Dunn’s tests.

**Liver and muscle expression of epigenetic machinery-related genes:** The expression of 15 genes encoding enzymes of the epigenetic machinery potentially involved in the observed changes in DNA methylation and histone marks (H3K9ac, H3K9me1 and H3K4me3) or known to be associated with metabolic disturbances in animals and humans was analyzed by RT-qPCR in liver and muscle tissues. The genes studied encoded DNA methyltransferases (*Dnmt1, Dnmt2, Dnmt3a, Dnmt3b, Dnmt3l*) and histone-modifying enzymes (*Suv39h1, Suv39h2, Set7/9, Jhdm2a, Hat1, Gcn5, Hdac5, Lsd1, Hdac3, Hdac7a*). Differences in gene expression are expressed as the ratio of expression between OP1 or OR1 and CD1 mice and between OP2 or OR2 and CD2 mice (Figures 9 and 10 and Table 2).

In both the liver and muscle, *Dnmt1, Dnmt3a* and *Dnmt3b* expression levels were similar in OP and OR mice born to both lean and obese mothers and their corresponding controls, CD1 and CD2. *Dnmt3b* expression was not detectable in muscle and *Dnmt3l* mRNA was barely detectable in either tissue. DNA hypomethylation was therefore not correlated with lower levels of *Dnmt1* or *Dnmt3a* and *Dnmt3b* expression after five months on a HFD. However, we cannot exclude the possibility that dysregulation of the production, activity or turnover of these DNMTs occurred earlier in the establishment of the response to the HFD and was subsequently normalized (Table 2).

Only *Dnmt2 (Trdmt1)* expression appeared to be modulated by the HFD in both liver and muscle. In the liver, *Dnmt2* expression was modulated only in the offspring of obese mothers (F2OM), being upregulated in both OP2 and OR2 mice (Figure 9A). In muscle, mirror-image patterns were observed for the expression of the DNA methyltransferase gene *Dnmt2,* which was found to have increased in OP1, OR1 and OR2 mice, but to have decreased in OP2 mice (Figure 9B). The role of *Dnmt2* remains a matter of debate, but a recent study highlighted its importance in the maintenance of genome stability, organ development, metabolic processes and resistance to oxidative stress [65]. Interestingly, *Dnmt2* was upregulated in tissues with normal levels of DNA methylation, but downregulated in the muscles of OP2 mice, which displayed DNA hypomethylation (Figure 5, Table 2).

In the liver, the expression of the two H3K9 methyltransferase genes, *Suv39h1* and *Suv39h2,* was upregulated only in OP1 mice (Figure 10), potentially inducing potential global changes in the level of H3K9 methylation, which is known to promote heterochromatin formation and gene silencing. However, as reported above, no difference in H3K9me3 level was found between the OP2, OR2, and CD2 groups. Nevertheless, we cannot exclude possible effects in the offspring of lean mothers (F1LM), in which it was not possible to study histone marks. (Table 2).

The H3K4 methyltransferase gene *Set7/9* was downregulated in the livers of both F1LM and F2OM mice. Despite this, F2OM mice had higher levels of H3K4me3 than controls, suggesting either a potent compensatory mechanism operating at the transcriptional level or the involvement of other molecules due to crosstalk between marks and enzymes. *Suv39h1* and *Set7/9* were upregulated in the muscles of both the OP1 and OR1 groups (Figure 10). In the offspring of obese mothers, *Suv39h1* was upregulated in OR2 mice only and *Set7/9* was regulated in opposite ways in OP2 and OR2 mice, tending to be upregulated in OR2 mice and significantly downregulated in OP2 mice. *Suv39h2* expression levels were high specifically in OR1 and OR2 mice. The *Set7/9* gene has been implicated in inflammation and diabetes, and its downregulation in monocytes has been shown to decrease the expression of key genes induced by inflammatory stimuli [66]. The expression of *Jhdm2a*, encoding an H3K9 demethylase, was weak only in OP2 mice. *Jhdm2a* is closely linked to several metabolic processes, as its product regulates the expression levels of genes encoding proteins involved in energy homeostasis, fat storage and glucose transport [67], [68] (Table 2).

Expression of the histone acetyl transferase gene *Hat1* was upregulated in the liver of OP2 mice only. Expression of the histone deacetylase gene *Hdac5* was significantly stronger in OR1 mice than in CD1 and OP1 mice, whereas no differences between groups were found in the offspring of obese mothers F2OM (Table 2). Interestingly, the *Hdac5* expression profile in muscle was similar to that in the liver, with specific upregulation in OR1 mice only, suggesting a role in obesity resistance specific to the offspring of lean mothers. Finally, the levels of expression of the *Gcn5* histone acetyl transferase gene *(Kat2a)* in the liver and of *Hat1 (Kat1)* in muscle were similar between groups. Expression of the histone deacetylase genes *Lsd1*, *Hdac3* and *Hdac7a* was not affected by the HFD or by susceptibility/resistance, in either the liver or muscle (data not shown).

Some tissue-specific expression patterns seemed to depend on the level of DNA methylation. In the liver, the hepatic expression of the H3K9 demethylase gene *Jhdm2a (Kdm3a)* was lower in both groups (OP2 and OR2) of offspring born to obese mothers F2OM than in the control. In muscle, normal DNA methylation was associated with the upregulation of *Suv39h1 (Kdm1a), Dnmt2 (Trdmt1)* and *Set7/9 (Kmt7*) in OP1 and OR2 mice. This upregulation contrasted with the lower levels of *Set7/9* and *Dnmt2* gene expression observed in the muscles of OP2 mice presenting DNA hypomethylation (Table 2).

In the liver, DNA methylation levels were low only in the OP1 group. By contrast, in muscle, DNA hypomethylation was observed in OP2 mice only. As far as the epigenetic machinery was concerned, histone K-methylation was affected in OP1 mice only, and histone K-acetylation was increased in OP2 muscle only (Table 2).

The lower levels of histone demethylation in the F2OM groups and the higher levels of histone K-deacetylation in OR1 mice probably reflect group-specific compensation mechanisms. It is therefore tempting to link DNA hypomethylation, lipotoxicity and tissue dysfunction. (Table 2).

The expression of genes encoding epigenetic machinery proteins involved in DNA methylation and histone K-deacetylation was altered in the livers of OR1 mice only. In OR mouse muscles, the only DNA methyltransferase gene displaying differential expression, *Dnmt2*, displayed opposite patterns of regulation in OR2 and OP2. The stronger expression of genes encoding proteins involved in histone K-methylation and histone K-deacetylation in OR1 and OR2 mice also suggests that specific epigenetic changes associated with either maternal leanness or obesity are involved in these processes of peripheral resistance. (Table 2).

Diet sensitivity was noted for genes or marks dysregulated in the same way in all groups fed the HFD. In the liver, the expression of *Set7/9* was clearly affected by diet, in both groups, as was histone H3K4me3 level.

The response to HFD and resistance/susceptibility to the obesogenic effects of the HFD were thus associated with tissue-specific changes in the expression of chromatin-modifying enzymes. It is difficult to determine the functional significance of these global changes, but these marks could potentially serve as markers for an effect of diet or for a susceptibility/resistance trait. Genome-wide analyses would make it possible to identify the sequences or genes on which these epigenetic modifiers act to affect susceptibility/resistance to the obesogenic effects of HFD. (Table 2).

DNA hypomethylation is a major feature of cells undergoing tumor progression and is a hallmark of many types of cancer, including hepatocellular carcinoma (HCC) in particular [69], [70]. It remains unclear whether HCC can develop from fatty liver, but it is striking that the risk of developing HCC is 4.5 times higher in obese individuals than in normal-weight subjects [71]. The DNA hypomethylation induced in DIO, with the development of hepatic steatosis, may therefore be a primary defect, as suggested by the dysregulation in the livers of OP mice of genes encoding proteins involved in liver cancer, with the potential to progress to more extensive cell damage and, potentially, carcinogenesis [72]. Thus, if this hypothesis can also be demonstrated in humans, DNA hypomethylation could be considered a marker of steatosis and, hence, of the risk of developing HCC. The association between DNA hypomethylation and NAFLD may provide new insight into the risk of HCC.

### Conclusion

Despite the well known deleterious effects of maternal obesity and T2D, the female offspring of obese mothers display greater resistance to the obesogenic effects of a HFD after weaning. Our results clearly demonstrate that, in a context of maternal obesity and diabetes, an appropriate dietary fatty-acid profile and intake during the periconceptional/gestation/lactation period helps the female offspring to cope with subsequent obesogenic conditions. These findings are reminiscent of those for other environmental factors, such as carbon tetrachloride or bile duct ligation triggering liver damage, and cocaine-self administration in rats [73], [74]. Fathers exposed to these chemicals display deleterious effects of exposure, whereas their offspring are better adapted to cope with future fibrogenic challenges to the liver or display a cocaine-resistant phenotype respectively. Exposure in the parent may therefore lead to greater resistance in the offspring. Exposure to a HFD leads to complex adaptation processes in several peripheral organs of the offspring, involving changes in the expression of imprinted genes and genes involved in metabolism and neurotransmission, together with more general epigenetic changes, such as marked changes in global DNA methylation, posttranslational histone modifications and the tissue-specific expression of genes encoding several chromatin-modifying enzymes. These findings highlight the complexity of the epigenetic mechanisms linked to overall adaptation to and attenuation of the deleterious programming effects of maternal obesity and T2D.

The networks identified highlight important connections between metabolism, neurotransmission and the imprinted gene network. The role of relevant metabolic pathways showing mirror-image patterns of expression in obesity-prone and obesity-resistant mice, or recruited for maternal effects specifically associated with either leanness or obesity/T2D and the unpredicted involvement of classes of liver genes related to neurotransmission reveal the importance of neural liver-brain crosstalk and provide new peripheral targets for prevention and treatment. We suggest that these gene networks may be considered as new environmental sensors and, hence, as novel, peripheral targets for the management of obesity and T2D. The different transcriptional and epigenetic modifications, according to maternal leanness or obesity/T2D status, underlying resistance or susceptibility to an obesogenic diet and associated with either the OP or OR phenotype therefore represent potential markers worthy of further study, with the aim of increasing our understanding of the etiology of T2D.

## Methods

### Animals and Diet

All experiments on animals were conducted after ethical review, under French government license, for three generations [21]. F0 generation*:* Male and female C57BL/6J mice were obtained from Harlan at four weeks of age. After one week of adaptation, the animals were housed in individual cages in a controlled-temperature room with a 12 h/12 h light/dark cycle and free access to water and a chow diet. F1LM generation: At six months of age, F0 males and F0 females were mated. The males were removed once the females were pregnant. The day after delivery, all F1LM litters were randomly reduced to four or five pups. After weaning, all mice, of both sexes, were transferred to individual cages and randomly assigned to the control (CD, 10% of calories from fat, 20% from protein and 70% from carbohydrates) or high-fat (HFD, 60% from fat, 20% from protein and 20% from carbohydrates) diets for 20 weeks. Diets were supplied in pellet form by Research Diets (CD: D12450B, HFD: D12492). F2 generation: At six months of age, F1LM female mice fed the HFD that became obese (OP1) were crossed with F1LM control male mice on the CD. The pregnant F1LM females were maintained on the CD from mating until the end of lactation and the F2OM offspring (male and female) was switched onto the HFD for 20 weeks after weaning. Mice were classified on the basis of their weight as sensitive (obesity-prone, OP1, OP2) or resistant (obesity-resistant, OR1, OR2) to the HFD [21]. In parallel, F1LM female mice on the CD (CD1) were crossed with F1LM control male mice to generate F2OM control mice fed the control diet after weaning (CD2). Reproducibility was ensured by carrying out three independent experiments with contemporary controls [21]. In these experiments, we were unable to obtain a sufficiently large number of pregnancies when females were maintained on a HFD, thus precluding comparisons with such a group.

### Caloric Intake

Food consumption was estimated by subtracting the amount of food left on the grid and the amount of spilled food from the initial weight of food supplied. Energy intake was then calculated, assuming 3.8 kcal/g for the CD and 5.2 kcal/g for the HFD.

### Body Composition

Body fat and lean mass were determined *in vivo* by dual-energy X-ray absorptiometry (DEXA) after 20 weeks on the CD or HFD. DEXA investigations in anesthetized animals (70 mg/kg ketamine/7 mg/kg xylazine) were performed on the whole body, excluding the head, with a Piximus apparatus® (Lunar Corporation, Madison, Wis., USA).

### Plasma Assays

Blood was collected after 20 weeks on the assigned diet. The animals were fasted for 6 h, and blood samples were then collected by retro-orbital sinus puncture. Glycemia, triglyceride concentration and total cholesterol and HDL cholesterol (HDL-C) concentrations were determined with a Behring RXL. Plasma insulin and leptin concentrations were determined with the Ultra-Sensitive Rat Insulin and Leptin ELISA kits, using the mouse standard, reference 90090 (Crystal Chem Inc). The HOMA index was calculated as the product of the fasting plasma insulin level (µU/ml) and the fasting plasma glucose level (mmol/l), divided by 22.5.

### Determination of the Fatty-acid Composition of LDL

Total fatty acids (FA) in LDL were analyzed by capillary gas chromatography on a Hewlett-Packard 5890 gas chromatograph attached to a 5971A mass detector (Hewlett-Packard), as previously described [75]. Total lipids were extracted from LDL as described by Folch *et al*. [76]. The extract was saponified by incubation at 60°C for 60 min with potassium hydroxide (13.2 g/l) and then esterified at 60°C for 60 min with boron trifluoride (BF3)-methanol, to generate fatty acid methyl esters. The chromatographic analysis was as described above for oxidized cholesterol derivatives, except for the oven temperature, which was increased from 220°C to 280°C at a rate of 10°C/min. Heptadecanoic acid (17∶0) was added to each sample as an internal standard before extraction, and the fatty-acid contents for saturated FA: palmitic acid (C16∶0), stearic acid (C18∶0); monounsaturated FA: palmitoleic acid (C16∶1), oleic acid (C18∶1); polyunsaturated n-6: linoleic acid (C18∶2), arachidonic acid (C20∶4); and for polyunsaturated n-3: docosahexaenoic acid (C22∶6), were determined from the ratio of the peak area of the sample to the peak area of the internal standard.

### Extraction of RNA and DNA

Total RNA was extracted from liver and adipose tissue with the RNeasy Mini kit® (Qiagen S.A). DNA was extracted from muscle, liver, kidneys, adipose tissue and testis with the DNeasy Blood and Tissue kit® (Qiagen S.A). DNA and RNA concentrations were determined with a NanoDrop ND-1000 spectrophotometer (NanoDrop Technologies) and quality was assessed by agarose gel electrophoresis.

### Custom-built Liver Microarray

The custom-built microarray design and array prehybridization and hybridization procedures have been described in detail elsewhere [23]. The microarray used contained 992 gene-specific 50-mer oligonucleotides generated from 63 imprinted genes (See Table S1), 256 genes encoding proteins involved in neurotransmission and 157 genes encoding proteins related to energy homeostasis. RNA quality was assessed by electrophoresis, in RNA 6000 assays on an Agilent 2100 bioanalyzer (Agilent Technologies). For cDNA synthesis, oligo-dT and RPO were added to 15 µg of total RNA and the mixture was incubated for 10 min at 70°C and then cooled on ice. We added 15 µl of 2.5×premix buffer (500 µM dCTP, 500 µM dATP, 500 µM dGTP, 100 µM dTTP, 100 µM Cy3-dUTP/Cy 5-dUTP, 600 U Superscript II Reverse Transcriptase, 10 mM DTT and 1×reverse transcriptase buffer), 1 µl of Cy3 dUTP or Cy5 dUTP and 2 µl of Superscript II reverse transcriptase to each sample. Reverse transcription was carried out overnight at 42°C. We then added 15 µl of 0.1 N NaOH and incubated the mixture for 10 min at 70°C to degrade the RNA template, and then with 15 µl of 0.1 N HCl to neutralize the reaction. The product was purified with the Qiaquick PCR Purification Kit® from Qiagen. A direct comparison strategy with dye-swapping was used to compare two conditions, and dye-swaps were replicated several times, with at least four animals/group. Comparisons between conditions were made according to an open-loop design, including the direct comparisons of major interest: OP1 *vs* CD1 (OP1/CD1); OP2 *vs* CD2 (OP2/CD2); OR2 *vs* CD2 (OR2/CD2); OR2 *vs* OP2 (OR2/OP2) and OP2 *vs* OP1 (OP2/OP1), such that secondary comparisons could be estimated [77]. Direct comparison between OR2 and OR1 mice was not possible due to the limited number of samples used for array hybridization. Arrays were normalized by LOESS regression, using the LIMMA library from the R/BIOCONDUCTOR packages to assess differential expression between conditions. Microarray experiments, described according to MIAME guidelines, have been deposited in NCBI’s Gene expression Omnibus repository under accession number GSE30085 (http://www.ncbi.nlm.nih.gov/geo/query/acc.cgi?acc=GSE30085).

### Microarray Analysis

Heat maps were constructed by compiling the differentially expressed genes (DEG) in the various comparisons (OP1/CD1, OP2/CD2, OR2/CD2, OR2/OP2, OP2/OP1), thereby allowing further indirect comparison between groups. Biologically relevant pathways were identified after importing Entrez Gene IDs into IPA software (version 8.7 Ingenuity® Systems, http://www.ingenuity.com). Several analyses were carried out with the lists of DEG obtained for the OR2/CD2, OR2/OP2 and OP2/OP1 comparisons and the subset of genes displaying dysregulation in both OP1/CD1 and OP2/CD2 comparisons. All the spot entries were recognized by the program and included in network and function/pathway analyses. The DEG were mapped to the genetic networks available in the Ingenuity database and were then ranked on the basis of their score. Only networks with scores of at least 20 were considered for interpretation, and functions and diseases were considered relevant if they had a *p* value<10^−5^.

### Reverse Transcription and Quantitative PCR (RT-qPCR)

First-strand cDNAs were synthesized from 2 µg of total RNA from adipose tissue samples, in the presence of 50 ng random hexamers (GE Healthcare), 400 nM dNTP and 200 U of Superscript™ II RNase H Reverse Transcriptase (Invitrogen), according to the manufacturer’s instructions. qPCR analyses were carried out with the Absolute Blue QPCR SYBR Green Rox Mix (Thermo Scientific), with a 7300 Real-Time PCR System (Applied Biosystems), according to the manufacturer’s instructions. Amplification was carried out in a final volume of 25 µl, with 2 x SYBR, 7.5 µM of each primer and 10 ng of the cDNA sample. Each reaction was run in duplicate. Eight-point standard curves were constructed from serial 10- fold dilutions, with cDNA pooled equally from all animals. *18S*, *Actb, Eif4a2, Sdha and Mrlp32* were tested as reference genes in the Genorm Software [78]. The qPCR were normalized with the Genorm normalization factor obtained with *Actb, Eif4a2* and *Sdha.* The differences in mRNA levels between CD and either OP or OR mice for each maternal effect (lean versus obese mother) were calculated and are expressed relative to CD levels. The complete list of primers used is provided in Table S4.

**Luminometric Methylation Assay (LUMA):** Global DNA methylation was analyzed as previously described by Karimi *et al*. [79]. Restriction enzymes (*Hpa*II, *Msp*I, and *Eco*RI) were purchased from New England Biolabs. Briefly, 500 ng of genomic DNA was subjected to double digestion with either *Hpa*II + *Eco*RI in buffer 1 (10 mM bis-Tris propane-HCl, 10 mM MgCl_2_, 1mM DTT) or *Msp*I + *Eco*RI in buffer 4 (50 mM potassium acetate, 20 mM Tris-acetate, 10 mM magnesium acetate, 1 mM DTT) for four hours at 37°C. The digested DNA was subjected to polymerase extension assays on the pyrosequencing platform. For this purpose, pyrosequencing annealing buffer (Biotage) was added to each reaction, and the reaction mixture was transferred to 24-well pyrosequencing plates and analyzed with PyroGold Q24 Reagents and the Pyromark^TM^Q24 Instrument, with an assay sequence defined as AC/TCGA. The level of cytosine methylation was finally determined by comparing the ratio of *Hpa*II to *Msp*I cleavages in the various samples.

**Histone preparation and western blotting:** Histones were prepared from approximately 50 mg of liver from 7 samples of each group of F2OM mice, as previously described [80]. Protein concentration was determined with a Pierce BCA assay kit (ThermoScientific). Histones (2 µg) were separated by electrophoresis in a 15% polyacrylamide SDS-PAGE gel and transferred onto PVDF membrane (ø 0.2 µm; Millipore). The membrane was blocked by incubation with 5% nonfat milk in PBS-Tween. After washing in PBS-Tween, membranes were incubated overnight with the primary antibody in 2% nonfat milk (anti-H3K9me3 1:1000, anti-H3K4me3 1:1000, anti-H3K9ac 1∶10000, anti-H3K9me1 1:1000; Millipore, 07-442, 07-473, 07-450, 07-352, respectively) and then for 1 hour with secondary antibody conjugated with horseradish peroxidase (anti-rabbit IgG 1∶10000, Sigma A6667). Signals were detected with ECL Plus (Amersham) and LAS-1000 (Fujifilm). The membranes were stripped and incubated for one hour with anti-pan H3 antibody (Abcam ab1791, 1∶5000). The secondary antibody and signal detection were as previously described. Signals were quantified with AIDA software and the signal for each specific modification was normalized to the amount of total H3. The whole western blotting procedure for each histone mark and total H3 was carried out twice. We then averaged the signals for the two experiments.

**Statistical analyses:** All statistical analyses were carried out with GraphPad Prism 2.0 Software. Phenotypic parameters (body weight, caloric intake, hormone levels) with a normal distribution were analyzed by one-way ANOVA, to test for differences between mice with lean and obese mothers and differences between diets. Bonferroni *post hoc* tests were carried out for pairwise comparisons. Fatty acid levels, IDEXA results, organ weights, RT-qPCR data and levels of DNA methylation and histone marks were analyzed by Kruskal-Wallis tests followed by Dunn's *post hoc* tests. Differences were considered to be significant if *p*<0.05.

## Supporting Information

Figure S1 **Validation, by RT-qPCR, of liver microarray data.** Plotting of the microarray data against RT-PCR values for the various ratios – OP1/CD1, OP2/CD2, OR2/CD2, OR2/OP2 and OP2/OP1 – for 15 different genes: *Acc1, Insr, Lepr, Ppara, Ppard, Rxr, Scd1, Thrb, Lpn1, Fabp2, Hnf4a, Ctcf, Xist, Gabrb3* and *Nnat*. The RT-qPCR and microarray values were consistent, for most of the 75 ratios, as indicated by the statistical significance of the linear regression equation R^2^ = 0.718, *p*<10^−5^).
(JPG)

Table S1 **List of imprinted genes.**
(XLS)

Table S2 **Top biological functions and diseases involving the genes differentially expressed between resistant OR2 and susceptible OP2 mice born to obese mothers (F2OM).** 142 genes identified as differentially regulated in the direct OR2/OP2 comparison were analyzed with IPA software.
(JPG)

Table S3 **IPA analysis of the subset of genes dysregulated in response to HFD in obese OP1 and OP2 mice.** After comparisons of the OP1/CD1 and OP2/CD2 series, 52 genes identified as dysregulated in both comparisons were subjected to IPA analysis. (A) Relevant diseases involving the genes deregulated in response to HFD in the liver of obese mice. The *p* values were calculated by IPA software for Fisher's exact tests. (B) Major networks of interaction clustering the genes deregulated in response to HFD in OP1 and OP2 livers.
(JPG)

Table S4 **List of qPCR primers.**
(XLS)

## References

[1] Barker DJ, Osmond C (1988) Low birth weight and hypertension. BMJ 297: 134–135. 10.1136/bmj.297.6641.134-b PMC1833827 3408942

[2] McMillen IC, Robinson JS (2005) Developmental origins of the metabolic syndrome: prediction, plasticity, and programming. Physiol Rev 85: 571–633. 15788706 10.1152/physrev.00053.2003

[3] Armitage JA, Taylor PD, Poston L (2005) Experimental models of developmental programming: consequences of exposure to an energy rich diet during development. J Physiol 565: 3–8. 15695245 10.1113/jphysiol.2004.079756 PMC1464498

[4] Nathanielsz PW, Poston L, Taylor PD (2007) In utero exposure to maternal obesity and diabetes: animal models that identify and characterize implications for future health. Obstet Gynecol Clin North Am 34: 201–212, vii–viii. 10.1016/j.ogc.2007.03.006 17572267

[5] Levin BE, Govek E (1998) Gestational obesity accentuates obesity in obesity-prone progeny. Am J Physiol 275: R1374–1379. 9756571 10.1152/ajpregu.1998.275.4.R1374

[6] Dabelea D, Hanson RL, Lindsay RS, Pettitt DJ, Imperatore G, et al (2000) Intrauterine exposure to diabetes conveys risks for type 2 diabetes and obesity: a study of discordant sibships. Diabetes 49: 2208–2211. 11118027 10.2337/diabetes.49.12.2208

[7] Boloker J, Gertz SJ, Simmons RA (2002) Gestational diabetes leads to the development of diabetes in adulthood in the rat. Diabetes 51: 1499–1506. 11978648 10.2337/diabetes.51.5.1499

[8] Gallou-Kabani C, Vige A, Gross MS, Rabes JP, Boileau C, et al (2007) C57BL/6J and A/J mice fed a high-fat diet delineate components of metabolic syndrome. Obesity (Silver Spring) 15: 1996–2005. 17712117 10.1038/oby.2007.238

[9] Dunn GA, Morgan CP, Bale TL (2010) Sex-specificity in transgenerational epigenetic programming. Horm Behav 59: 290–295. 20483359 10.1016/j.yhbeh.2010.05.004

[10] Gabory A, Attig L, Junien C (2009) Sexual Dimorphism in Environmental Epigenetic Programming. Molecular cellular endocrinology 25: 8–18. 10.1016/j.mce.2009.02.015 19433243

[11] Giraudo SQ, Della-Fera MA, Proctor L, Wickwire K, Ambati S, et al (2010) Maternal high fat feeding and gestational dietary restriction: effects on offspring body weight, food intake and hypothalamic gene expression over three generations in mice. Pharmacol Biochem Behav 97: 121–129. 20430050 10.1016/j.pbb.2010.04.017

[12] Srinivasan M, Katewa SD, Palaniyappan A, Pandya JD, Patel MS (2006) Maternal high-fat diet consumption results in fetal malprogramming predisposing to the onset of metabolic syndrome-like phenotype in adulthood. Am J Physiol Endocrinol Metab 291: E792–799. 16720630 10.1152/ajpendo.00078.2006

[13] Smith J, Cianflone K, Biron S, Hould FS, Lebel S, et al (2009) Effects of maternal surgical weight loss in mothers on intergenerational transmission of obesity. J Clin Endocrinol Metab 94: 4275–4283. 19820018 10.1210/jc.2009-0709

[14] Kral JG, Biron S, Simard S, Hould FS, Lebel S, et al (2006) Large maternal weight loss from obesity surgery prevents transmission of obesity to children who were followed for 2 to 18 years. Pediatrics 118: e1644–1649. 17142494 10.1542/peds.2006-1379

[15] Burdge GC, Lillycrop KA (2010) Nutrition, Epigenetics, and Developmental Plasticity: Implications for Understanding Human Disease. Ann Rev Nutr 30: 1–7. 20415585 10.1146/annurev.nutr.012809.104751

[16] Torrens C, Brawley L, Anthony FW, Dance CS, Dunn R, et al (2006) Folate supplementation during pregnancy improves offspring cardiovascular dysfunction induced by protein restriction. Hypertension 47: 982–987. 16585422 10.1161/01.HYP.0000215580.43711.d1

[17] Boujendar S, Arany E, Hill D, Remacle C, Reusens B (2003) Taurine supplementation of a low protein diet fed to rat dams normalizes the vascularization of the fetal endocrine pancreas. J Nutr 133: 2820–2825. 12949371 10.1093/jn/133.9.2820

[18] Dolinoy DC, Weidman JR, Waterland RA, Jirtle RL (2006) Maternal genistein alters coat color and protects Avy mouse offspring from obesity by modifying the fetal epigenome. Environ Health Perspect 114: 567–572. 16581547 10.1289/ehp.8700 PMC1440782

[19] Waterland RA, Travisano M, Tahiliani KG (2007) Diet-induced hypermethylation at agouti viable yellow is not inherited transgenerationally through the female. Faseb J 21: 3380–3385. 17551099 10.1096/fj.07-8229com

[20] Lillycrop KA, Phillips ES, Jackson AA, Hanson MA, Burdge GC (2005) Dietary protein restriction of pregnant rats induces and folic acid supplementation prevents epigenetic modification of hepatic gene expression in the offspring. J Nutr 135: 1382–1386. 15930441 10.1093/jn/135.6.1382

[21] Gallou-Kabani C, Vige A, Gross MS, Boileau C, Rabes JP, et al (2007) Resistance to high-fat diet in the female progeny of obese mice fed a control diet during the periconceptual, gestation, and lactation periods. Am J Physiol Endocrinol Metab 292: E1095–1100. 17164437 10.1152/ajpendo.00390.2006

[22] Gabory A, Roseboom TJ, Moore T, Moore LG, Junien C (2013) Placental contribution to the origins of sexual dimorphism in health and diseases: sex chromosomes and epigenetics Biol Sex Differ Mar 21;4(1): 5. [Epub ahead of print]. 10.1186/2042-6410-4-5 PMC3618244 23514128

[23] Vige A, Gallou-Kabani C, Gross MS, Fabre A, Junien C, et al (2006) An oligonucleotide microarray for mouse imprinted genes profiling. Cytogenet Genome Res 113: 253–261. 16575188 10.1159/000090840

[24] Waterland RGC (2002) Potential for metabolic imprinting by nutritional perturbation of epigenetic gene regulation. Public health issues in infant and child nutrition 48: 317.

[25] Tycko B, Morison IM (2002) Physiological functions of imprinted genes. J Cell Physiol 192: 245–258. 12124770 10.1002/jcp.10129

[26] Gallou-Kabani C, Gabory A, Tost J, Karimi M, Mayeur S, et al (2010) Sex- and Diet-Specific Changes of Imprinted Gene Expression and DNA Methylation in Mouse Placenta under a High-Fat Diet. PLoS One 5: e14398. 21200436 10.1371/journal.pone.0014398 PMC3006175

[27] Puschel GP (2004) Control of hepatocyte metabolism by sympathetic and parasympathetic hepatic nerves. Anat Rec A Discov Mol Cell Evol Biol 280: 854–867. 15382015 10.1002/ar.a.20091

[28] Uyama N, Geerts A, Reynaert H (2004) Neural connections between the hypothalamus and the liver. Anat Rec A Discov Mol Cell Evol Biol 280: 808–820. 15382020 10.1002/ar.a.20086

[29] Yi CX, la Fleur SE, Fliers E, Kalsbeek A (2010) The role of the autonomic nervous liver innervation in the control of energy metabolism. Biochim Biophys Acta 1802: 416–431. 20060897 10.1016/j.bbadis.2010.01.006

[30] Bruce KD, Cagampang FR, Argenton M, Zhang J, Ethirajan PL, et al (2009) Maternal high-fat feeding primes steatohepatitis in adult mice offspring, involving mitochondrial dysfunction and altered lipogenesis gene expression. Hepatology 50: 1796–1808. 19816994 10.1002/hep.23205

[31] Oben JA, Mouralidarane A, Samuelsson AM, Matthews PJ, Morgan ML, et al (2010) Maternal obesity during pregnancy and lactation programs the development of offspring non-alcoholic fatty liver disease in mice. J Hepatol 52: 913–920. 20413174 10.1016/j.jhep.2009.12.042

[32] Kalsbeek A, La Fleur S, Van Heijningen C, Buijs RM (2004) Suprachiasmatic GABAergic inputs to the paraventricular nucleus control plasma glucose concentrations in the rat via sympathetic innervation of the liver. J Neurosci 24: 7604–7613. 15342726 10.1523/JNEUROSCI.5328-03.2004 PMC6729629

[33] Xie H, Lautt WW (1996) Insulin resistance caused by hepatic cholinergic interruption and reversed by acetylcholine administration. Am J Physiol 271: E587–592. 8843755 10.1152/ajpendo.1996.271.3.E587

[34] Latour MG, Lautt WW (2002) The hepatic vagus nerve in the control of insulin sensitivity in the rat. Auton Neurosci 95: 125–130. 11871777 10.1016/s1566-0702(01)00390-3

[35] Vatamaniuk MZ, Horyn OV, Vatamaniuk OK, Doliba NM (2003) Acetylcholine affects rat liver metabolism via type 3 muscarinic receptors in hepatocytes. Life Sci 72: 1871–1882. 12586224 10.1016/s0024-3205(02)02506-7

[36] Kamiji MM, Inui A (2007) Neuropeptide y receptor selective ligands in the treatment of obesity. Endocr Rev 28: 664–684. 17785427 10.1210/er.2007-0003

[37] Hoffstedt J, Arner E, Wahrenberg H, Andersson DP, Qvisth V, et al (2010) Regional impact of adipose tissue morphology on the metabolic profile in morbid obesity. Diabetologia 53: 2496–2503. 20830466 10.1007/s00125-010-1889-3

[38] Wang ET, Sandberg R, Luo S, Khrebtukova I, Zhang L, et al (2008) Alternative isoform regulation in human tissue transcriptomes. Nature 456: 470–476. 18978772 10.1038/nature07509 PMC2593745

[39] Pan Q, Shai O, Lee LJ, Frey BJ, Blencowe BJ (2008) Deep surveying of alternative splicing complexity in the human transcriptome by high-throughput sequencing. Nat Genet 40: 1413–1415. 18978789 10.1038/ng.259

[40] Pembrey M (1996) Imprinting and transgenerational modulation of gene expression; human growth as a model. Acta Genet Med Gemellol (Roma) 45: 111–125. 8872020 10.1017/s0001566000001197

[41] Beaudet AL, Jiang YH (2002) A rheostat model for a rapid and reversible form of imprinting-dependent evolution. Am J Hum Genet 70: 1389–1397. 11992247 10.1086/340969 PMC379123

[42] Gallou-Kabani C, Junien C (2005) Nutritional epigenomics of metabolic syndrome: new perspective against the epidemic. Diabetes 54: 1899–1906. 15983188 10.2337/diabetes.54.7.1899

[43] Weksberg R, Shuman C, Caluseriu O, Smith AC, Fei YL, et al (2002) Discordant KCNQ1OT1 imprinting in sets of monozygotic twins discordant for Beckwith-Wiedemann syndrome. Hum Mol Genet 11: 1317–1325. 12019213 10.1093/hmg/11.11.1317

[44] Joe MK, Lee HJ, Suh YH, Han KL, Lim JH, et al.. (2008) Crucial roles of neuronatin in insulin secretion and high glucose-induced apoptosis in pancreatic beta-cells. Cell Signal. 10.1016/j.cellsig.2008.01.005 18289831

[45] Waterland RA, Garza C (2002) Early postnatal nutrition determines adult pancreatic glucose-responsive insulin secretion and islet gene expression in rats. J Nutr 132: 357–364. 11880555 10.1093/jn/132.3.357

[46] Chen M, Gavrilova O, Liu J, Xie T, Deng C, et al (2005) Alternative Gnas gene products have opposite effects on glucose and lipid metabolism. Proc Natl Acad Sci U S A 102: 7386–7391. 15883378 10.1073/pnas.0408268102 PMC1129092

[47] Weinstein LS, Liu J, Sakamoto A, Xie T, Chen M (2004) Minireview: GNAS: normal and abnormal functions. Endocrinology 145: 5459–5464. 15331575 10.1210/en.2004-0865

[48] Kelly ML, Moir L, Jones L, Whitehill E, Anstee QM, et al (2009) A missense mutation in the non-neural G-protein alpha-subunit isoforms modulates susceptibility to obesity. Int J Obes (Lond) 33: 507–518. 19238158 10.1038/ijo.2009.30

[49] Gabory A, Ripoche MA, Le Digarcher A, Watrin F, Ziyyat A, et al (2009) H19 acts as a trans regulator of the imprinted gene network controlling growth in mice. Development 136: 3413–3421. 19762426 10.1242/dev.036061

[50] Varrault A, Gueydan C, Delalbre A, Bellmann A, Houssami S, et al (2006) Zac1 regulates an imprinted gene network critically involved in the control of embryonic growth. Dev Cell 11: 711–722. 17084362 10.1016/j.devcel.2006.09.003

[51] Fauque P, Ripoche MA, Tost J, Journot L, Gabory A, et al.. (2010) Modulation of imprinted gene network in placenta results in normal development of in vitro manipulated mouse embryos. Hum Mol Genet. 10.1093/hmg/ddq059 20150233

[52] Sugden MC, Holness MJ (2006) Mechanisms underlying regulation of the expression and activities of the mammalian pyruvate dehydrogenase kinases. Arch Physiol Biochem 112: 139–149. 17132539 10.1080/13813450600935263

[53] Koza RA, Nikonova L, Hogan J, Rim JS, Mendoza T, et al (2006) Changes in gene expression foreshadow diet-induced obesity in genetically identical mice. PLoS Genet 2: e81. 16733553 10.1371/journal.pgen.0020081 PMC1464831

[54] Kozak LP, Newman S, Chao PM, Mendoza T, Koza RA (2010) The early nutritional environment of mice determines the capacity for adipose tissue expansion by modulating genes of caveolae structure. PLoS One 5: e11015. 20574519 10.1371/journal.pone.0011015 PMC2888576

[55] Verrier L, Vandromme M, Trouche D (2011) Histone demethylases in chromatin cross-talks. Biol Cell 103: 381–401. 21736555 10.1042/BC20110028

[56] Bartke T, Vermeulen M, Xhemalce B, Robson SC, Mann M, et al (2010) Nucleosome-interacting proteins regulated by DNA and histone methylation. Cell 143: 470–484. 21029866 10.1016/j.cell.2010.10.012 PMC3640253

[57] Gabory A, Attig L, Junien C (2011) Epigenetic mechanisms involved in developmental nutritional programming. World J Diabetes 2: 164–175. 22010058 10.4239/wjd.v2.i10.164 PMC3196195

[58] Macfarlane DP, Zou X, Andrew R, Morton NM, Livingstone DE, et al (2011) Metabolic pathways promoting intrahepatic fatty acid accumulation in methionine and choline deficiency: implications for the pathogenesis of steatohepatitis. Am J Physiol Endocrinol Metab 300: E402–409. 21119028 10.1152/ajpendo.00331.2010 PMC3043621

[59] Pogribny IP, Tryndyak VP, Muskhelishvili L, Rusyn I, Ross SA (2007) Methyl deficiency, alterations in global histone modifications, and carcinogenesis. J Nutr 137: 216S–222S. 17182829 10.1093/jn/137.1.216S

[60] James SJ, Pogribny IP, Pogribna M, Miller BJ, Jernigan S, et al (2003) Mechanisms of DNA damage, DNA hypomethylation, and tumor progression in the folate/methyl-deficient rat model of hepatocarcinogenesis. J Nutr 133: 3740S–3747S. 14608108 10.1093/jn/133.11.3740S

[61] Miao F, Wu X, Zhang L, Yuan YC, Riggs AD, et al (2007) Genome-wide analysis of histone lysine methylation variations caused by diabetic conditions in human monocytes. J Biol Chem 282: 13854–13863. 17339327 10.1074/jbc.M609446200

[62] Miao F, Wu X, Zhang L, Riggs AD, Natarajan R (2008) Histone methylation patterns are cell-type specific in human monocytes and lymphocytes and well maintained at core genes. J Immunol 180: 2264–2269. 18250434 10.4049/jimmunol.180.4.2264 PMC2683787

[63] Siebel AL, Fernandez AZ, El-Osta A (2010) Glycemic memory associated epigenetic changes. Biochem Pharmacol 80: 1853–1859. 20599797 10.1016/j.bcp.2010.06.005

[64] Villeneuve LM, Kato M, Reddy MA, Wang M, Lanting L, et al. Enhanced levels of microRNA-125b in vascular smooth muscle cells of diabetic db/db mice lead to increased inflammatory gene expression by targeting the histone methyltransferase Suv39h1. Diabetes 59: 2904–2915. 10.2337/db10-0208 PMC2963550 20699419

[65] Schaefer M, Lyko F (2010) Solving the Dnmt2 enigma. Chromosoma 119: 35–40. 19730874 10.1007/s00412-009-0240-6

[66] Li Y, Reddy MA, Miao F, Shanmugam N, Yee JK, et al (2008) Role of the histone H3 lysine 4 methyltransferase, SET7/9, in the regulation of NF-kappaB-dependent inflammatory genes. Relevance to diabetes and inflammation. J Biol Chem 283: 26771–26781. 18650421 10.1074/jbc.M802800200 PMC2546554

[67] Inagaki T, Tachibana M, Magoori K, Kudo H, Tanaka T, et al (2009) Obesity and metabolic syndrome in histone demethylase JHDM2a-deficient mice. Genes Cells 14: 991–1001. 19624751 10.1111/j.1365-2443.2009.01326.x

[68] Tateishi K, Okada Y, Kallin EM, Zhang Y (2009) Role of Jhdm2a in regulating metabolic gene expression and obesity resistance. Nature 458: 757–761. 19194461 10.1038/nature07777 PMC4085783

[69] Lin CH, Hsieh SY, Sheen IS, Lee WC, Chen TC, et al (2001) Genome-wide hypomethylation in hepatocellular carcinogenesis. Cancer Res 61: 4238–4243. 11358850

[70] Tangkijvanich P, Hourpai N, Rattanatanyong P, Wisedopas N, Mahachai V, et al (2007) Serum LINE-1 hypomethylation as a potential prognostic marker for hepatocellular carcinoma. Clin Chim Acta 379: 127–133. 17303099 10.1016/j.cca.2006.12.029

[71] Caldwell SH, Crespo DM, Kang HS, Al-Osaimi AM (2004) Obesity and hepatocellular carcinoma. Gastroenterology 127: S97–103. 15508109 10.1053/j.gastro.2004.09.021

[72] Suzuki H, Toyota M, Sato H, Sonoda T, Sakauchi F, et al (2006) Roles and causes of abnormal DNA methylation in gastrointestinal cancers. Asian Pac J Cancer Prev 7: 177–185. 16839207

[73] Zeybel M, Hardy T, Wong YK, Mathers JC, Fox CR, et al (2012) Multigenerational epigenetic adaptation of the hepatic wound-healing response. Nat Med 18: 1369–1377. 22941276 10.1038/nm.2893 PMC3489975

[74] Vassoler FM, White SL, Schmidt HD, Sadri-Vakili G, Pierce RC (2013) Epigenetic inheritance of a cocaine-resistance phenotype. Nat Neurosci 16: 42–47. 23242310 10.1038/nn.3280 PMC3531046

[75] Viens L, Athias A, Lizard G, Simard G, Gueldry S, et al (1996) Effect of lipid transfer activity and lipolysis on low density lipoprotein (LDL) oxidizability: evidence for lipolysis-generated non-esterified fatty acids as inhibitors of LDL oxidation. J Lipid Res 37: 2179–2192. 8906595

[76] Folch J, Lees M, Sloane Stanley GH (1957) A simple method for the isolation and purification of total lipides from animal tissues. J Biol Chem 226: 497–509. 13428781

[77] Kerr MK, Churchill GA (2001) Experimental design for gene expression microarrays. Biostatistics 2: 183–201. 12933549 10.1093/biostatistics/2.2.183

[78] Vandesompele J, De Preter K, Pattyn F, Poppe B, Van Roy N, et al (2002) Accurate normalization of real-time quantitative RT-PCR data by geometric averaging of multiple internal control genes. Genome Biol 3: 34.01–34.11. 10.1186/gb-2002-3-7-research0034 PMC126239 12184808

[79] Karimi M, Johansson S, Stach D, Corcoran M, Grander D, et al (2006) LUMA (LUminometric Methylation Assay)–a high throughput method to the analysis of genomic DNA methylation. Exp Cell Res 312: 1989–1995. 16624287 10.1016/j.yexcr.2006.03.006

[80] Shechter D, Dormann HL, Allis CD, Hake SB (2007) Extraction, purification and analysis of histones. Nat Protoc 2: 1445–1457. 17545981 10.1038/nprot.2007.202

