# Supplementary figures and images for: Dietary Alleviation of Maternal Obesity and Diabetes: Increased Resistance to Diet-Induced Obesity Transcriptional and Epigenetic Signatures
Source: PLoS One. 2013 Jun 24;8(6):e66816. doi: 10.1371/journal.pone.0066816 (PMC3691260; doi:10.1371/journal.pone.0066816)

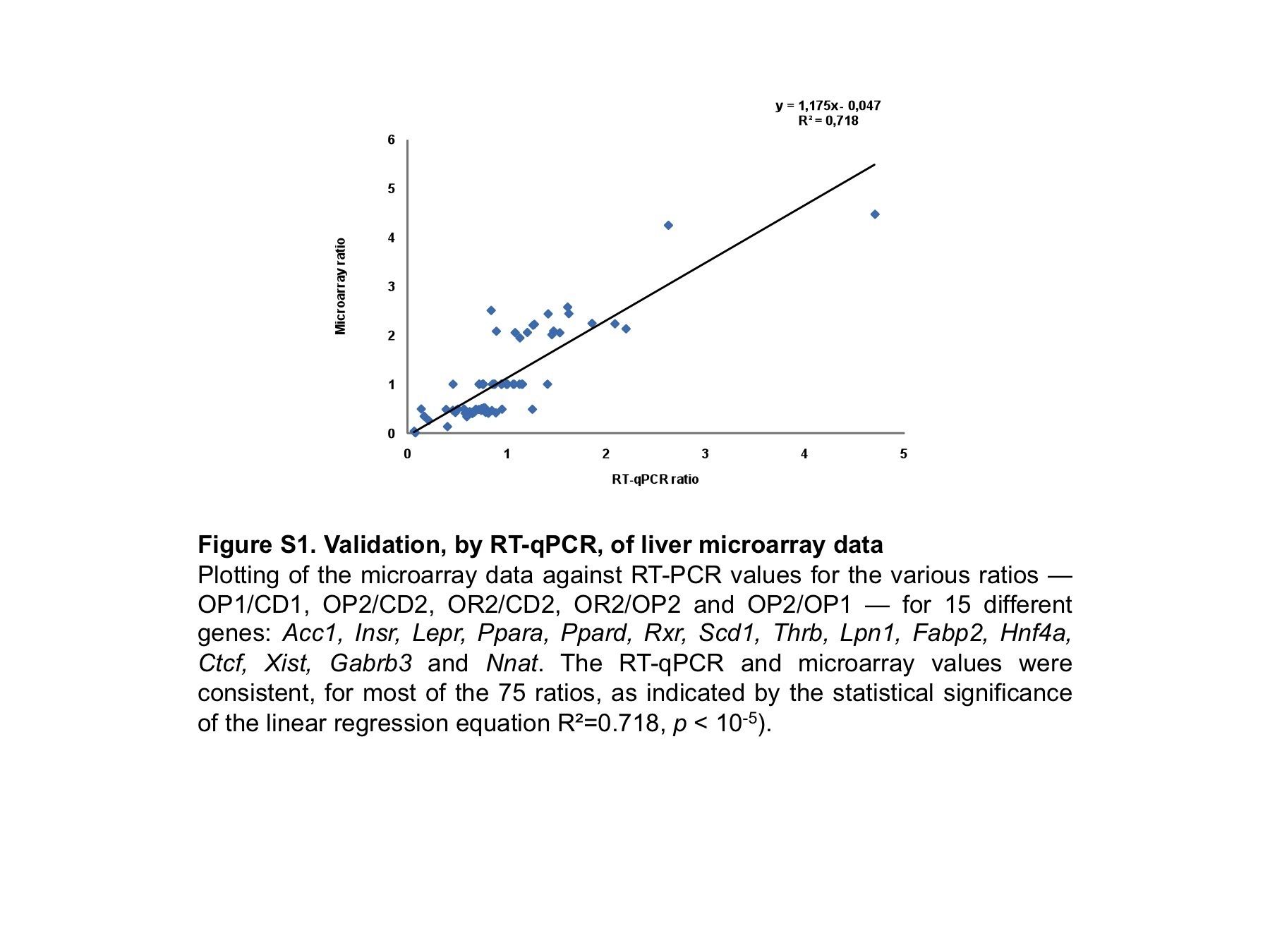

Supplement: Figure S1 — Validation, by RT-qPCR, of liver microarray data. Plotting of the microarray data against RT-PCR values for the various ratios – OP1/CD1, OP2/CD2, OR2/CD2, OR2/OP2 and OP2/OP1 – for 15 different genes: Acc1, Insr, Lepr, Ppara, Ppard, Rxr, Scd1, Thrb, Lpn1, Fabp2, Hnf4a, Ctcf, Xist, Gabrb3 and Nnat. The RT-qPCR and microarray values were consistent, for most of the 75 ratios, as indicated by the statistical significance of the linear regression equation R2 = 0.718, p<10−5). (JPG) [file pone.0066816.s001.jpg]

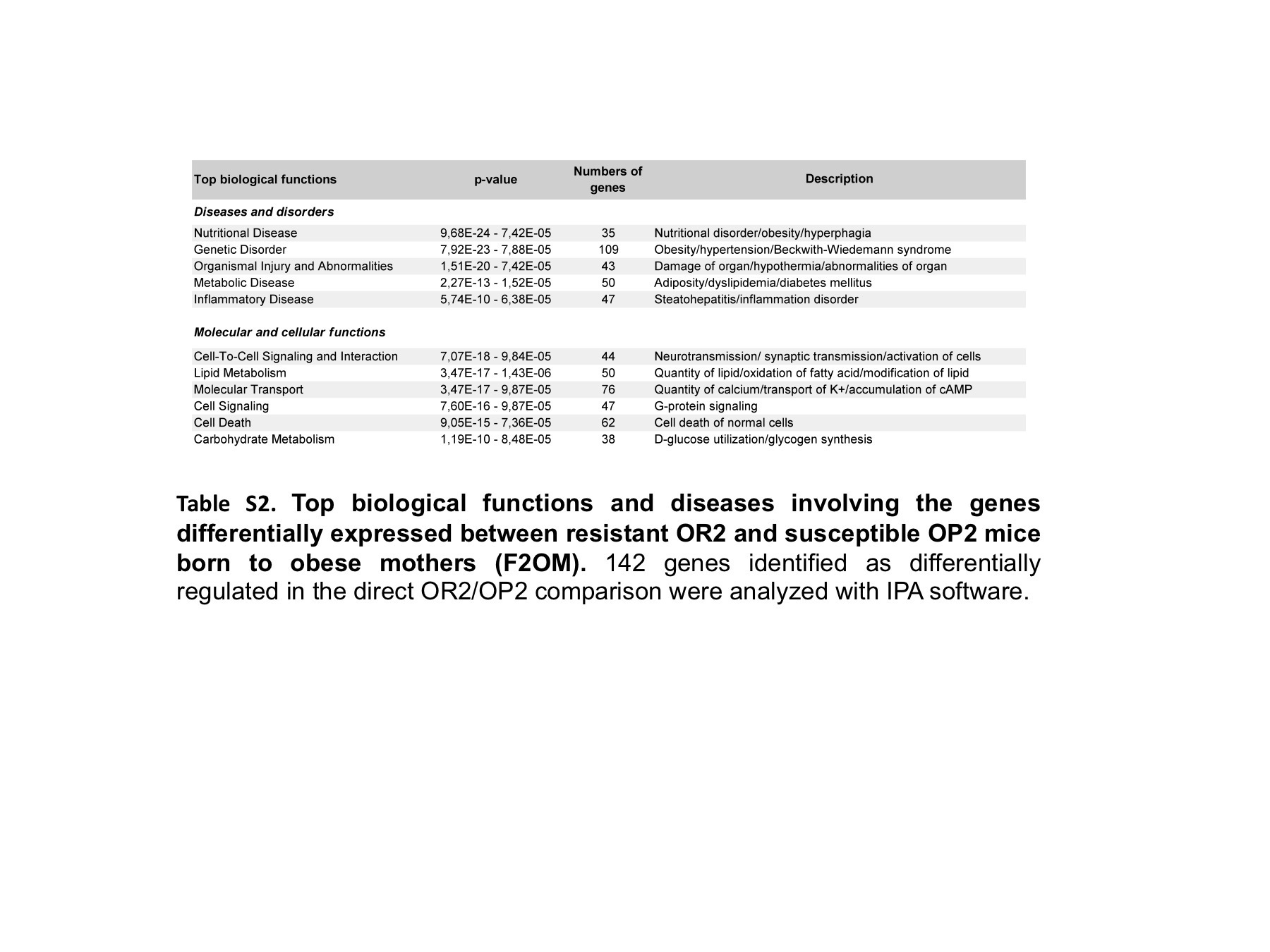

Supplement: Table S2 — Top biological functions and diseases involving the genes differentially expressed between resistant OR2 and susceptible OP2 mice born to obese mothers (F2OM). 142 genes identified as differentially regulated in the direct OR2/OP2 comparison were analyzed with IPA software. (JPG) [file pone.0066816.s003.jpg]

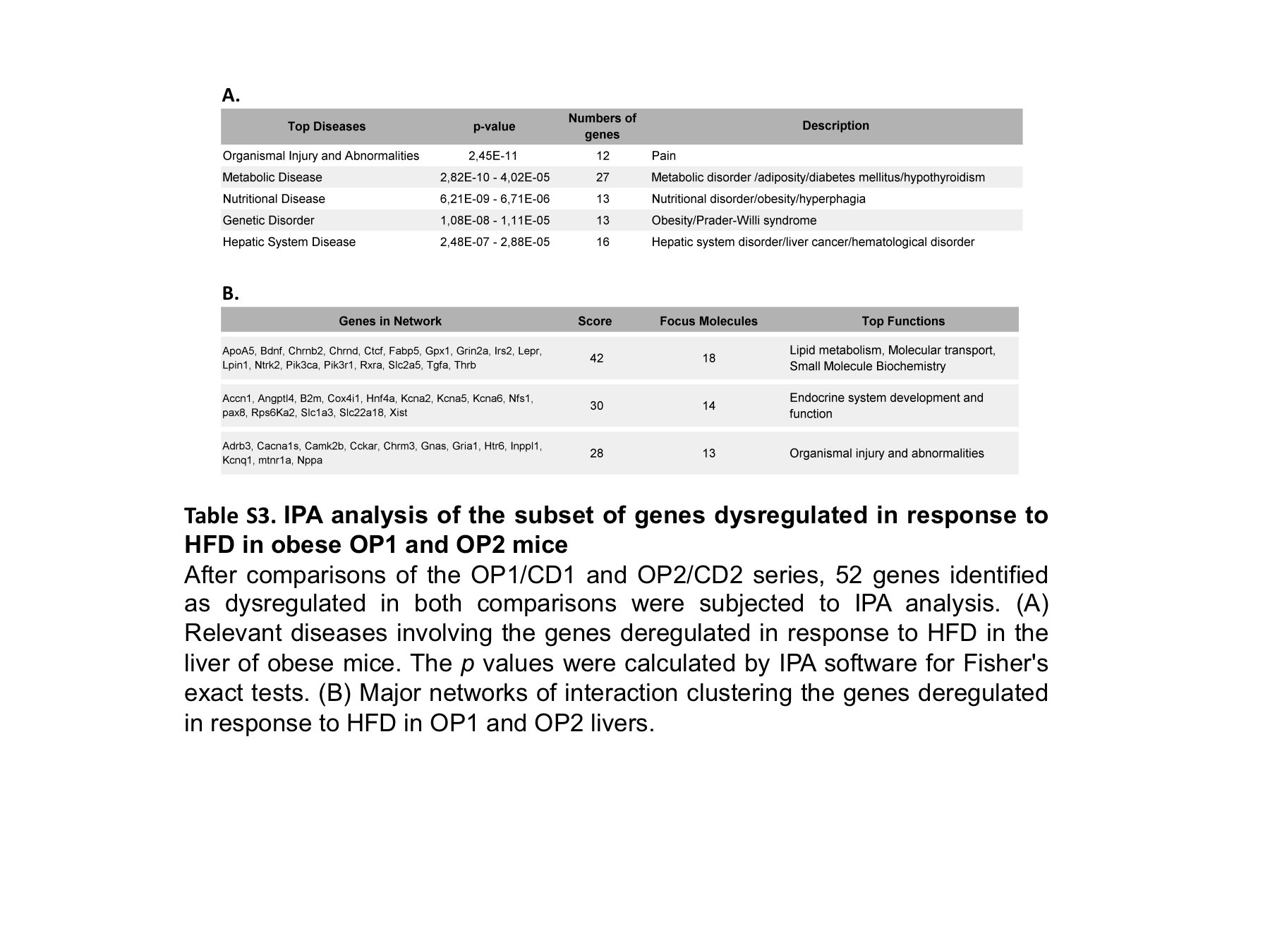

Supplement: Table S3 — IPA analysis of the subset of genes dysregulated in response to HFD in obese OP1 and OP2 mice. After comparisons of the OP1/CD1 and OP2/CD2 series, 52 genes identified as dysregulated in both comparisons were subjected to IPA analysis. (A) Relevant diseases involving the genes deregulated in response to HFD in the liver of obese mice. The p values were calculated by IPA software for Fisher's exact tests. (B) Major networks of interaction clustering the genes deregulated in response to HFD in OP1 and OP2 livers. (JPG) [file pone.0066816.s004.jpg]
